# Imputation Without Doing Imputation: A New Method for the Detection of Non-Genotyped Causal Variants

**DOI:** 10.1002/gepi.21792

**Published:** 2014-02-17

**Authors:** Richard Howey, Heather J Cordell

**Affiliations:** Institute of Genetic Medicine, Newcastle University, International Centre for Life, Central ParkwayNewcastle upon Tyne, United Kingdom

**Keywords:** haplotype analysis, imputation, GWAS

## Abstract

Genome-wide association studies allow detection of non-genotyped disease-causing variants through testing of nearby genotyped SNPs. This approach may fail when there are no genotyped SNPs in strong LD with the causal variant. Several genotyped SNPs in weak LD with the causal variant may, however, considered together, provide equivalent information. This observation motivates popular but computationally intensive approaches based on imputation or haplotyping. Here we present a new method and accompanying software designed for this scenario. Our approach proceeds by selecting, for each genotyped “anchor” SNP, a nearby genotyped “partner” SNP, chosen via a specific algorithm we have developed. These two SNPs are used as predictors in linear or logistic regression analysis to generate a final significance test. In simulations, our method captures much of the signal captured by imputation, while taking a fraction of the time and disc space, and generating a smaller number of false-positives. We apply our method to a case/control study of severe malaria genotyped using the Affymetrix 500K array. Previous analysis showed that fine-scale sequencing of a Gambian reference panel in the region of the known causal locus, followed by imputation, increased the signal of association to genome-wide significance levels. Our method also increases the signal of association from 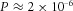 to 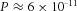. Our method thus, in some cases, eliminates the need for more complex methods such as sequencing and imputation, and provides a useful additional test that may be used to identify genetic regions of interest.

## Introduction

Over the last 5–10 years, genome-wide association studies (GWAS) have proved a popular and highly successful approach for identifying genomic regions (loci) that harbor single-nucleotide polymorphisms (SNPs) associated with complex diseases [Visscher et al., 2012]. Early GWAS used microarray-based genotyping technologies to survey between 100,000 and 500,000 SNPs across the genome; subsequent technological developments have led to the routine use of genotyping arrays containing anywhere between 500,000 and 4.3 million markers. SNPs present on genotyping arrays were initially chosen based on surveys of known human genetic variation such as HapMap [The International HapMap Consortium, 2003] and tended to be heavily focused toward SNPs present in European populations. Commonly used genotyping arrays typically provided lower levels of coverage of genetic variation in non-European populations [Barrett and Cardon, 2006], particularly African populations that generally show lower levels of linkage disequilibrium (LD) owing to their population histories. The inclusion of larger numbers of more varied populations in large-scale sequencing projects such as 1000 Genomes Project [1000 Genomes Project Consortium et al., 2012], which have informed the development of newer genotyping arrays, has redressed this balance somewhat, but it is still true to say that not every population's genetic variation will be equally well “tagged” [Barrett and Cardon, 2006; Johnson et al., 2001] by currently available genotyping arrays.

The utility of a GWAS is premised on the idea that a nongenotyped disease-causing variant can be detected through testing a nearby genotyped SNP that is in strong LD with the causal variant. For populations whose genetic variation is not well tagged, this premise may fail if the disease-causing variant is neither present, nor highly correlated with a variant present, on the genotyping array used. Use of more sophisticated analysis approaches based on *imputation* [Browning and Browning, 2007b; Howie et al., 2009, 2012; Li and Abecasis, 2006; Li et al., 2010; Marchini et al., 2007; Servin and Stephens, 2007] or *haplotyping* [Allen and Satten, 2009a, 2009b; Browning and Browning, 2007a, 2007b, 2008; Gusev et al., 2011], rather than single-SNP testing, can allow the recovery of information at causal variants that are well-tagged by *combinations* of genotyped SNPs rather than by any individual genotyped SNP. However, these approaches tend to be highly computer-intensive and may require extensive postanalysis quality control to produce reliable results [Browning and Browning, 2008; Howie et al., 2012]. It is probably true to say that the main use of imputation over the last few years has been to conveniently generate common panels of SNPs (genotyped and imputed), in order to enable large-scale meta-analyses of studies that have been carried out using different genotyping arrays [Band et al., 2013; Berndt et al., 2013; Zeggini et al., 2008], rather than, as originally conceived, to improve the signal of association at poorly-tagged causal variants per se.

Here we describe a new GWAS method and software implementation, SnipSnip, that is specifically designed to improve the signal of association at poorly tagged causal variants and to increase power over standard single-SNP analysis in situations where there are a number of SNPs in low LD with the causal variant. The method proceeds by selecting, for each genotyped *anchor* SNP, a nearby genotyped *partner* SNP (chosen from a window of SNPs surrounding the anchor SNP). The partner SNP is selected on the basis of a specific algorithm we have developed, which uses the correlation between the two SNPs to construct a score in which higher scoring potential partner SNPs are expected to be more “useful.” See Methods for details of how we define “useful.” These two SNPs are then used as predictors in a linear or logistic regression analysis to generate a final *artificial-imputation* (AI) significance test for the anchor SNP. The procedure is repeated for every genotyped anchor SNP across the genome.

## Methods

### The Artifical-Imputation Test

Two different logistic regression models are used in the construction of the AI test. Let *p* be the probability an individual is diseased, then association between disease status and genotypes at the anchor and partner SNPs can be modelled by a logistic regression model:

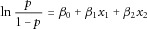
1where *x*_1_ and *x*_2_ are the number of minor alleles at the anchor and partner SNPs respectively (assuming an additive allelic model on the log odds scale, equivalent to a multiplicative model on the odds scale, between and within loci) and β_0_, β_1_, and β_2_ are regression coefficients (to be estimated). For a set of cases and controls the likelihood function is given by:

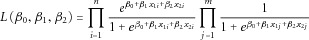
2where *i* and *j* index cases and controls (with *n* and *m* the number of cases and controls), respectively. This model is compared with the logistic regression model for the partner SNP only:

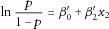
3where 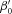 and 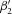 are (new) regression coefficients.

The AI test uses a likelihood ratio test to compare models 1 and 3, giving a χ^2^ test statistic on one degree of freedom:

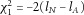
4where 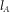 and 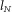 are the log-likelihoods from alternative and null models 1 and 2, maximized with respect to their regression coefficients, respectively. The corresponding *P*-value gives the significance of the anchor SNP *conditional* on the partner SNP. That is, we test whether the additional information from the anchor SNP improves the signal of association (with disease status) over that provided by the partner SNP alone. If the local LD pattern is such that the anchor and partner SNPs provide different information concerning a causal variant, then this situation (of improved significance) may be expected to occur.

We also considered an alternative test that evaluated the significance of adding an anchor-partner SNP interaction term to model 1 as considered by Slavin et al. [2011] and Wei et al. [2013] (motivated by the observation that such a “local” interaction could correspond to a haplotype effect marking a single untyped causal variant, see Gyenesei et al. [2012]). However, we found no circumstances where this test was superior to our original test (results not shown).

A similar AI test can be constructed for quantitative trait phenotypes instead of case/control status. In this case, the two following linear regression models are compared:

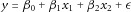
5

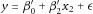
6where *y*, *x*_1_, *x*_2_, and ε are the quantitative trait value, number of minor alleles at the anchor and partner SNPs, and error term, respectively. The error terms are assumed to be normally distributed. The test statistic is given by a 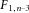 test statistic (where *n* is the number of subjects), which compares the residual sum of squares from the fitted linear models.

### Selection of the Partner SNP

The partner SNP is chosen from a window of potential partner SNPs surrounding the anchor SNP on the genome. The SNP window is defined by a fixed number of SNPs to the left and right of the anchor SNP (except when the anchor SNP is near the end of a chromosome, in which case there may be more potential partner SNPs on one side than the other). A SNP window of size *w* corresponds to considering 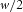 SNPs to the left and 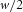 SNPs to the right of the anchor SNP. An alternative way to define the SNP window is by base pair position units, where any SNP within a certain base pair position distance of the anchor SNP is included in the SNP window. However, use of a fixed SNP window size is generally preferred (see Results).

Each potential partner SNP within the window is assigned a score that depends on the observed correlation between the anchor SNP and the potential partner SNP. We use a predefined metric (see below) to map the Pearson's *r*^2^ correlation coefficient (as defined by Wellek and Ziegler [2009]) between the two SNPs to a score between 0 and 100, where higher values are considered better. The potential partner SNP with the highest score is then selected to be the final partner SNP. In the event of a tie, the SNP showing the highest score that appears first in the SNP window is chosen. Correlation is calculated using the entire dataset regardless of phenotype (i.e., all cases and all controls, for a case/control dataset, or all individuals for a dataset with quantitative trait measurements). This feature ensures that the procedure for choosing the partner SNP is statistically independent of the subsequent AI test performed.

Our goal here is to choose the partner SNP that is most likely to provide a *useful* test result. We define useful in this context as the ability to detect a causal variant when single-SNP GWAS methods fail. In particular, we are interested in obtaining improved performance for our method over standard single-SNP testing in regions of low LD and poorly tagged causal variants. Intuitively, we would expect that that a partner SNP that has “too high” a correlation with the anchor SNP would not be that useful for the AI test, as the anchor SNP is unlikely to provide much extra information for association testing compared to that provided by the partner alone. Equally, a partner SNP that has “too low” a correlation with the anchor SNP will not be that useful, as it does not add any information compared to that provided by the anchor alone, and so the AI test will not be very different from the anchor's single-SNP test. Therefore, our goal is to pick a partner whose correlation with the anchor SNP is not “too high” and not “too low.” Naïvely, we may think it would be a good idea simply to choose the SNP that gives the most significant AI test result as the partner SNP. However, this approach would inflate significance levels across all anchor SNPs, resulting in an invalid test. Instead we use a simulation procedure (see below) to precompute the metric that is used to select the partner SNP in such a way that higher scores are assigned to correlation patterns that tend to lead to higher significance for the AI test than for single-SNP testing, when there is a causal variant in the vicinity of the two SNPs.

We note that other measures could be used instead of, or in addition to, the correlation between the two SNPs, when choosing the appropriate partner SNP for each anchor SNP. A detailed investigation of possible alternative measures would be an interesting topic for future work. For example, one could make use of alternative LD measures such as 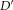. In this spirit, we initially investigated the use of a more complicated haplotype-based metric to choose between potential partner SNPs. The disadvantage of both the haplotype-based metric and 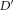 is that these approaches are computationally less efficient, as haplotypes need to be resolved (estimated) in order to calculate the metric and thus choose the best partner SNP, whereas this is not required for our current correlation-based metric (*r*^2^ can be calculated on the basis of the unphased genotype data alone, see Wellek and Ziegler [2009]). Investigation of our proposed haplotype-based metric did not find its performance to be superior (in terms of power or type I error) to that of our correlation-based metric (data not shown), and thus, for the time being, we have not considered it any further.

### Correlation Metric Construction

It is not obvious which anchor-partner SNP correlations are most useful. In order to determine this, we used an empirical approach to sample from the entire sample space of possible anchor-disease-partner SNP haplotype configurations. Our reasoning was that the performance of both single-SNP tests and our proposed AI test will be largely determined by the population haplotype frequencies at these three SNPs. By sampling from this haplotype space, we could investigate which haplotype configurations result in situations where the AI test has improved power over single-SNP testing, and what resulting correlations between the anchor and partner SNP are induced in these situations. We note that, in practice, the utility of the AI test (in comparison to single-SNP testing) will be lowest when the disease SNP is already well tagged by the anchor SNP. If the disease and anchor SNP are highly correlated (as would be expected when using dense genotyping arrays that have been specifically designed to tag the population under study), then we would expect single-SNP tests to perform well and no improvement to be seen from use of more complicated methods such as imputation, AI or haplotype testing. This does not concern us as we are not proposing AI as an *alternative* to single-SNP testing, rather we propose it as a useful *additional* test that can, in some circumstances, detect a complementary signal. We are therefore not overly concerned about situations where single-SNP testing already works well, we simply want to ensure that our correlation metric performs well in situations where there is, in fact, a complementary signal to be found.

We thus performed one million computer simulations to calculate, for different haplotype configurations (resulting in different anchor-partner SNP correlations) the resulting AI and single-SNP logistic regression tests. In each simulation replicate, a random haplotype frequency partition was chosen to model the haplotype frequencies at three SNPs (the anchor SNP, a disease-causing SNP, and the partner SNP). The eight haplotype frequencies were randomly simulated as follows:

1. The eight haplotype frequencies were initially undefined over the unit interval.
2. A random haplotype was picked from the remaining undefined haplotype frequencies (with equal probability).
3. A frequency for the haplotype was picked from a uniform distribution scaled from 0 to 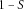, where *S* is the sum of the previously defined haplotype frequencies.
4. Steps 2 and 3 were repeated until all the haplotype frequencies were defined, with the last haplotype frequency taking the value remaining within the unit interval.

Once the eight haplotype frequencies had been assigned, genotype data for 1,000 cases and 1,000 controls were simulated using these haplotype frequencies and assuming multiplicative penetrance values of 0.01, 0.015 and 0.0225 for genotypes containing 0, 1, and 2 causal alleles, respectively. From each simulation replicate the following results were recorded: (i) the anchor-partner SNP correlation using all case/control data; (ii) the AI test statistic; and (iii) the single-SNP logistic regression test statistic at the anchor SNP.

To determine the usefulness of a given correlation, the differences between the test statistics were calculated by subtracting the single-SNP logistic regression test statistic from the AI test statistic. A threshold was set to select the top 1% of these differences. The correlations were then divided into bins of size 0.05. For each correlation bin the probability of the test statistic differences being in the top 1% was calculated using all simulated anchor-partner SNP correlations falling in that bin. That is, given a correlation in a certain correlation bin, we estimated the probability that the corresponding test statistic difference is in the top 1%. A map was then constructed to convert the anchor-partner SNP correlations to a score by using the midpoints of the bins and the calculated probabilities to fit a continuous curve, with additional points specified at (0, 0) and (1, 0). A polynomial of degree 5 was fitted to the points (excluding the three lowest and highest correlation values, which were fitted separately, as the tails do not follow the general pattern). The resultant curve and calculated points are shown in supplementary Figure S1 (leftmost panel), scaled so that the maximum of the curve takes value 100. The top anchor-partner scores are seen to lie within the correlation interval [0.257, 0.357], suggesting that anchor-partner SNP combinations whose correlation lies within this range are most useful for the AI test, consistent with our intuition that we should seek a partner SNP whose correlation is not “too high” and not “too low” with the anchor SNP.

The same procedure was repeated using different sets of multiplicative penetrances and for different numbers of cases and controls, with no discernable difference between the resultant curves. Decreasing the top percentage test statistic difference threshold used to construct the curve (to a value lower than 1%) resulted in a shift of the curve peak toward 0.25 (i.e., toward lower correlations). However, lower correlations are also more likely to give a nonsignificant AI test result. There is a trade-off between: (i) a low chance of finding a partner SNP with a possibly very significant AI test result; and (ii) a higher chance of finding a suitable partner SNP but with a possibly lower AI test result. We deemed the top 1% threshold to be a sensible choice in practice.

We also constructed similar correlation maps under the assumption of dominant and recessive penetrances at the causal SNP. The resultant curves are shown in supplementary Figure S1. The top anchor-partner SNP scores lie within the correlation intervals [0.356, 0.456] and [0.376, 0.476] for the dominant and recessive metrics respectively. It should be noted that multiplicative (on the odds scale) logistic regression models were still used when calculating the AI test, as use of dominant and recessive regression models in general reduces the power of the AI test (see Results).

We note that the algorithm used to choose the partner SNP is statistically independent of the subsequent AI test carried out using these two SNPs. (Effectively, the choice of partner SNP is determined by the correlations in genotype observed between the two SNPs, while the subsequent AI test is determined by their mutual correlation with phenotype). Thus, we expect the type I error rate of the overall procedure to be comparable to that of a standard single-SNP GWAS in which we analyse each genotyped SNP individually; we do not expect the type I error rate to be inflated on account of the search procedure used to choose the partner SNPs. Simulations (see Results) suggest that this expectation is indeed correct.

### Implementation

The AI test has been implemented in C++ in an accompanying software package, SnipSnip, which is freely available from http://www.staff.ncl.ac.uk/richard.howey/snipsnip/. Execution times are favorable, even when single-SNP analyses are also performed. For example, for 2,118 subjects and 17,515 SNPs, analysis using a 10-SNP window without inclusion of covariates takes around 17 sec in SnipSnip (compared to 30 sec in PLINK [Purcell et al., 2007]) when the linear regression version is used, or 8 sec in SnipSnip (compared to 33 sec in PLINK) when the logistic regression version is used. (Timing comparisons performed in Linux on Six-Core AMD OpteronTM Processors with 2.6 GHz CPUs, using 64 bit versions of SnipSnip and PLINK).

## Results

### Evaluation of AI Method Using Simulated Data

**Figure 1:**
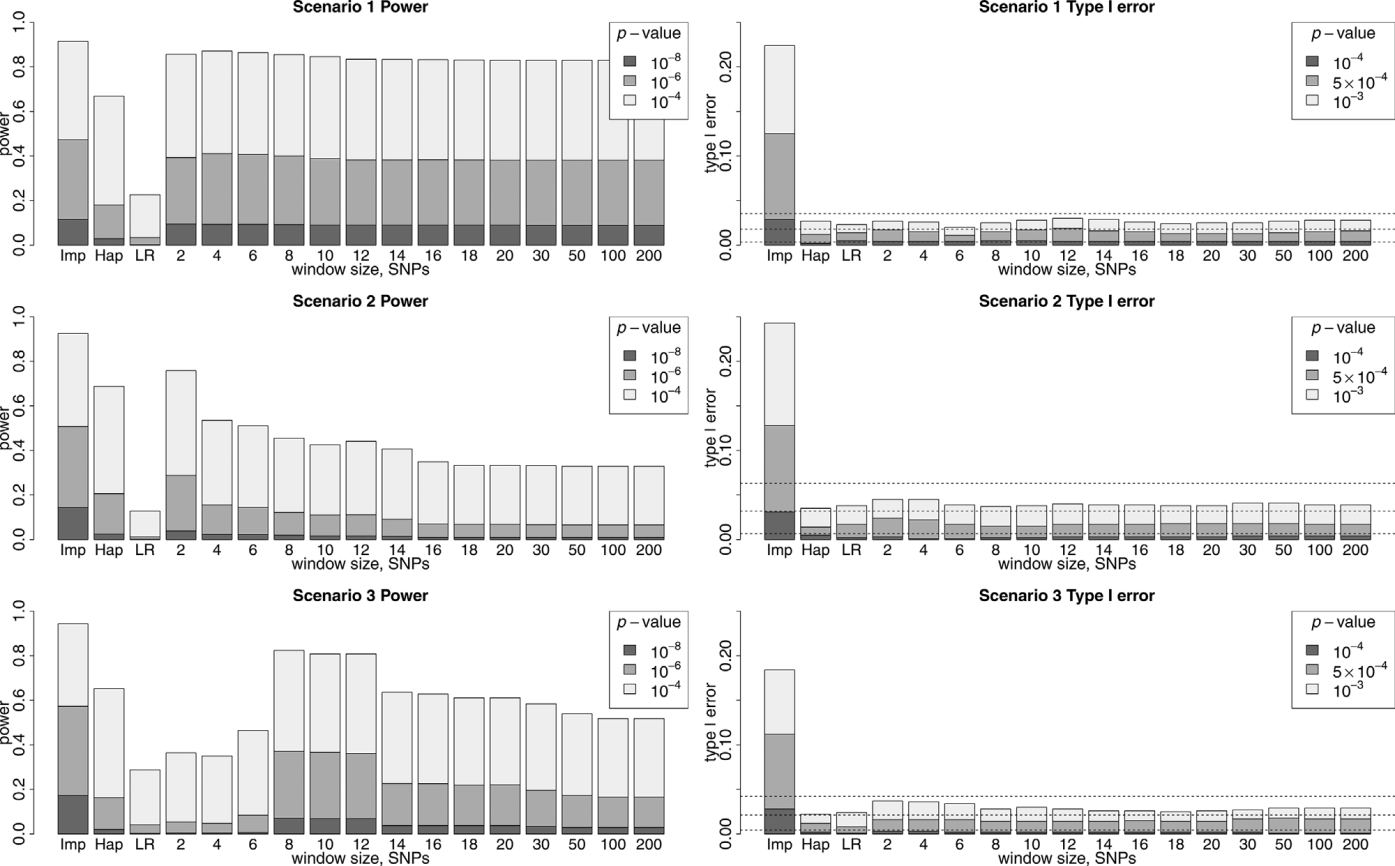
Powers and type I errors for Scenarios 1–3, windows defined by number of SNPs. Shown are bar plots of the calculated powers (for *P*-values 10^−8^, 10^−6^, and 10^−4^) and type I errors (for *P*-values 10^−4^, 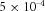 and 10^−3^) for Scenarios 1–3 for imputation (Imp), haplotype analysis (Hap), single-SNP logistic regression (LR), and the AI test with different SNP window sizes. The standard multiplicative model and correlation metrics were used in the AI test. Rows 1-3 show Scenarios 1-3, respectively.

We used computer simulations to evaluate the performance of our proposed AI test and to compare it to other approaches. Figure 1 (left hand panels) shows a comparison of the powers to achieve various *P*-values in three different simulation scenarios for imputation, haplotype analysis, single-SNP logistic regression in PLINK, and the AI test (with the AI test taking different SNP window sizes, see Methods) respectively. Imputation was carried out using the program IMPUTE2 [Howie et al., 2009; Marchini et al., 2007] with prephasing [Howie et al., 2012] in SHAPEIT [Delaneau et al., 2012], using data from the 1000 Genomes Project [1000 Genomes Project Consortium et al., 2012] (Phase I interim data, updated release April 2012) as a reference panel. Haplotype analysis was carried out using the haplotype regression approach implemented in the program UNPHASED [Dudbridge, 2008; Dudbridge et al., 2011], using a sliding window of 5-SNP haplotypes.

The different scenarios considered corresponded to “causal” SNPs located in different regions on chromosome 11. Specifically, the causal SNPs were: Scenario 1: rs240686 at Build 36 base pair (BP) position 63989461; Scenario 2: rs10501081 at Build 36 BP position 27192637; Scenario 3: rs9804546 at Build 36 BP position 7088763. In each scenario the genotype relative risks were assumed to be 1.5 and 2.25 for possession of one and two risk alleles at the causal SNP, respectively. (We note that an allelic relative risk of 1.5 is probably an upper limit for the kind of effect sizes generally observed in GWAS; however, we do not expect our results—in terms of the comparison between different methods—to be overly sensitive to our choice of this value. The use of smaller relative risks—with the same sample size—would simply result in lower powers for all methods considered, while the use of smaller relative risks but with larger sample sizes— as are generally, in fact, used in GWAS—would result in similar or increased powers for all methods, depending on the precise effect size and sample size chosen).

Detection power was defined as the proportion of replicates in which we detected a SNP (or the middle SNP of a 5-SNP haplotype) lying within 110 kb of the causal SNP, with “detection” corresponding to the SNP or haplotype achieving a nominal *P*-value of 10^−8^, 10^−6^, or 10^−4^, respectively. Any detected SNP lying within 110 kb of the causal SNP was thus considered as a true-positive. To evaluate type I error, data were simulated under the null hypothesis by assuming genotype relative risks of 1.0 and 1.0 for possession of one and two risk alleles, respectively, with (false) detections of any SNPs lying within 110 kb of the “null” causal SNP calculated at nominal *P*-value thresholds 10^−4^, 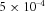, and 10^−3^.

Figure 1 (left hand panels) shows that the AI test clearly outperforms single-SNP logistic regression in these scenarios, regardless of window size, capturing part (although not all) of the signal that is achievable through use of imputation, and often outperforming haplotype analysis (depending on the window size chosen). The different window sizes in AI do show different patterns of detection power for the different scenarios. In Scenario 1, it can be seen that window size does not make any discernable difference to the detection power of the AI test. This is due to an anchor SNP having a nearby SNP that gives a significant result when picked as the partner SNP, but also a very good correlation score (see Methods), ensuring it remains chosen as the window size increases. In Scenario 2, the optimal combination of anchor and partner SNP does not have a favorable correlation score when compared to other possible partner SNPs, and thus the detection power decreases as the window size increases. This highlights the fact that the correlation score is only a rough guide as to which SNP is most likely to be the optimal partner SNP. In Scenario 3, it can be seen that the SNP window size must be increased to eight SNPs for the AI test to achieve maximum detection power, after which the detection power decreases as the SNP window size increases. This is due to the best partner SNP occurring 4 SNPs from the anchor SNP, with worse-performing partner SNPs (but with better correlation scores) not appearing until seven SNPs from the anchor SNP.

The optimal SNP window size for AI is clearly dependent on the data, and so cannot be known in advance. Different SNP window sizes could be tried but this would raise a multiple testing issue. If the SNP window size is too large, then there is a greater chance of picking a poorly performing partner SNP. This is because, if the anchor and partner SNP are far apart, they are less likely to be both correlated with a causal variant but may still achieve a high correlation score. Conversely, if the SNP window is too small, then we do not allow a sufficient choice of partner SNPs as to pick the optimal one. A compromise has to be made between these extremes. We suggest using a SNP window size of 10 SNPs (and probably no more than 20 SNPs), which seems to provide a reasonable compromise in practice.

Figure 1 (right hand panels) shows the proportion of replicates showing type I errors in the different scenarios for imputation, haplotype analysis, single-SNP logistic regression, and the AI test using different SNP window sizes. Because we define “detection” here as any SNP that lies within 110 kb of the causal SNP achieving a nominal *P*-value of 10^−4^, 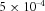, or 10^−3^, respectively, for single-SNP logistic regression, haplotype analysis, and the AI test we expect the overall probability of type I error to correspond to *a* times the nominal *P*-value, where *a* is the number of tested anchor SNPs (or haplotypes) within the 110 kb detection region (36, 65, and 43 SNPs for scenarios 1, 2, and 3, respectively), assuming independence of the *a* tests. These expected proportions of type I errors are shown as dashed lines. The observed probabilities of type I error for AI, single-SNP logistic regression and haplotype analysis lie, as expected, mostly below these lines because the tested anchor SNPs (or haplotypes) are not completely independent, and so use of a Bonferroni correction to account for multiple testing of *a* anchor SNPs is overly conservative. The type I error probabilities thus achieve their desired levels and do not appear to be overly sensitive to the choice of SNP window size.

**Figure 2:**
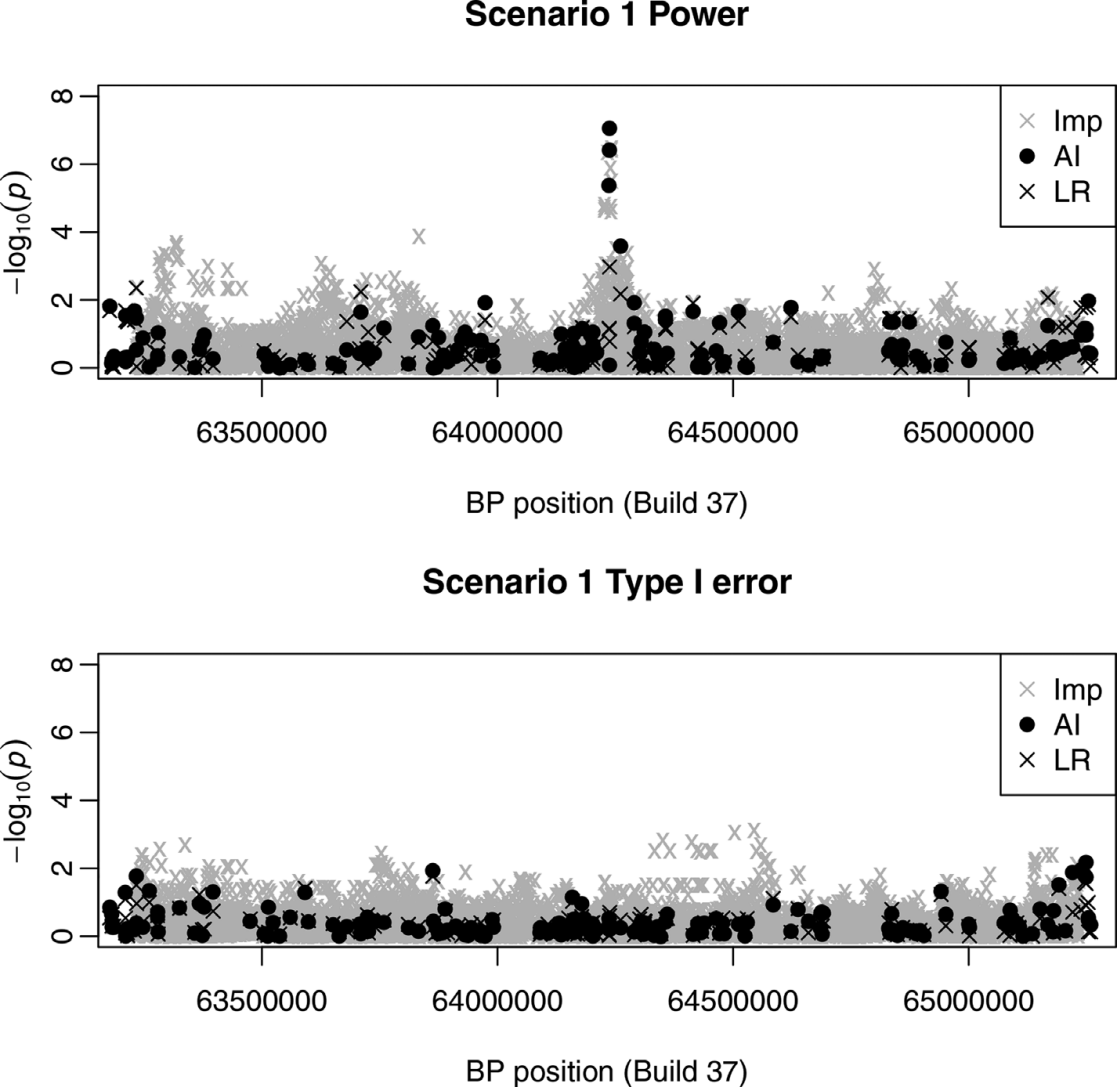
Results from a single power replicate (top plot) and a single type I error replicate (bottom plot) of Scenario 1. Gray crosses show the results obtained from imputation (Imp), black dots show the results obtained from AI and black crosses show the results obtained from single-SNP logistic regression (LR) analysis in PLINK.

Interestingly, Figure 1 shows that, with imputation, the proportion of replicates showing type I errors is much larger than with single-SNP logistic regression, haplotype analysis, and AI. Thus, although the left hand panels of Figure 1 suggest that imputation has generally slightly higher detection power than AI, this higher detection power is achieved at the expense of a higher *number* (although not necessarily a higher *rate*) of type I errors. We attribute this phenomenon to the larger multiple testing burden that is incurred when carrying out imputation, on account of larger number of SNPs that are imputed (and therefore tested). To illustrate this phenomenon, we present in Figure 2 the results seen within a single simulation replicate of power (top plot) or type I error (bottom plot). In the power replicate, both imputation and AI are successful in detecting the simulated disease SNP. However, in both the power replicate and the type I error replicate, the much larger number of tests performed when using imputation results in a much higher likelihood of observing a false detection (at any given significance level) within any region, compared to single-SNP analysis, or AI, even though we anticipate that the nominal (i.e., per-SNP) type I error rate for all three methods should be the same.

The logical consequence of this observation is that the threshold for declaring “genome-wide significance” when carrying out an imputation-based GWAS should be higher than when carrying out a standard (genotyped SNPs only) GWAS, if one wishes to maintain the same number of false-positives (or overall probability of making any false-positive detections) as is incurred in a standard GWAS. The precise threshold to be used will depend on how many SNPs have been successfully imputed (and are thus tested) and the degree of LD between them; visually, in this example, it appears that increasing the required 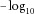
(*P*-value) threshold by one unit would achieve approximately the right level. Taking this “rule-of-thumb,” we should therefore logically compare the powers to detect *P*-values of 10^−8^, 10^−6^, or 10^−4^ using AI with the powers to detect *P*-values of 10^−9^, 10^−7^, or 10^−5^ using imputation, if we are to be comparing “like with like.” Applying this idea to our current results, we find this has the effect of reducing the detection power of imputation (at *P*-value thresholds 10^−9^, 10^−7^, or 10^−5^) in these scenarios to a value similar to that of AI (at *P*-value thresholds 10^−8^, 10^−6^, or 10^−4^), while maintaining a comparable number of type I errors (data not shown).

To our knowledge, it has not generally been appreciated that use of imputation may require the use of a more stringent threshold for declaring genome-wide significance, at least when using standard frequentist tests of association. Most studies using genome-wide imputation (e.g., Ripke et al. [2013]) have used the same thresholds for highlighting “suggestive” and “significant” findings as were used in the pre-imputation era, when tests in GWAS were carried out at genotyped SNPs only. Although not, strictly speaking, “correct,” in practice this is unlikely to have caused any serious increase in false-positive findings on account of the fact that most “suggestive” and “significant” findings from a primary GWAS analysis are taken forward for replication in independent studies. Thus, any additional false-positives arising at the primary stage are likely to be removed at the replication stage. However, it does highlight the importance of comparing like with like (in terms of expected numbers of type I errors) when comparing the relative detection powers of different methods.

Supplementary Figure S2 shows the detection powers and type I errors of the AI test compared to imputation and haplotype analysis when the SNP window sizes are defined using BP distances rather than numbers of SNPs. The patterns are very similar to those seen in Figure 1, although we do find slightly lower powers than when using a fixed number of SNPs to define the window size. For this reason, we prefer to use a fixed number of SNPs. The number of SNPs in the window will obviously vary depending on the number of genotyped SNPs in the region; for samples genotyped on a very dense SNP array the use of BP distances may be preferred.

**Figure 3:**
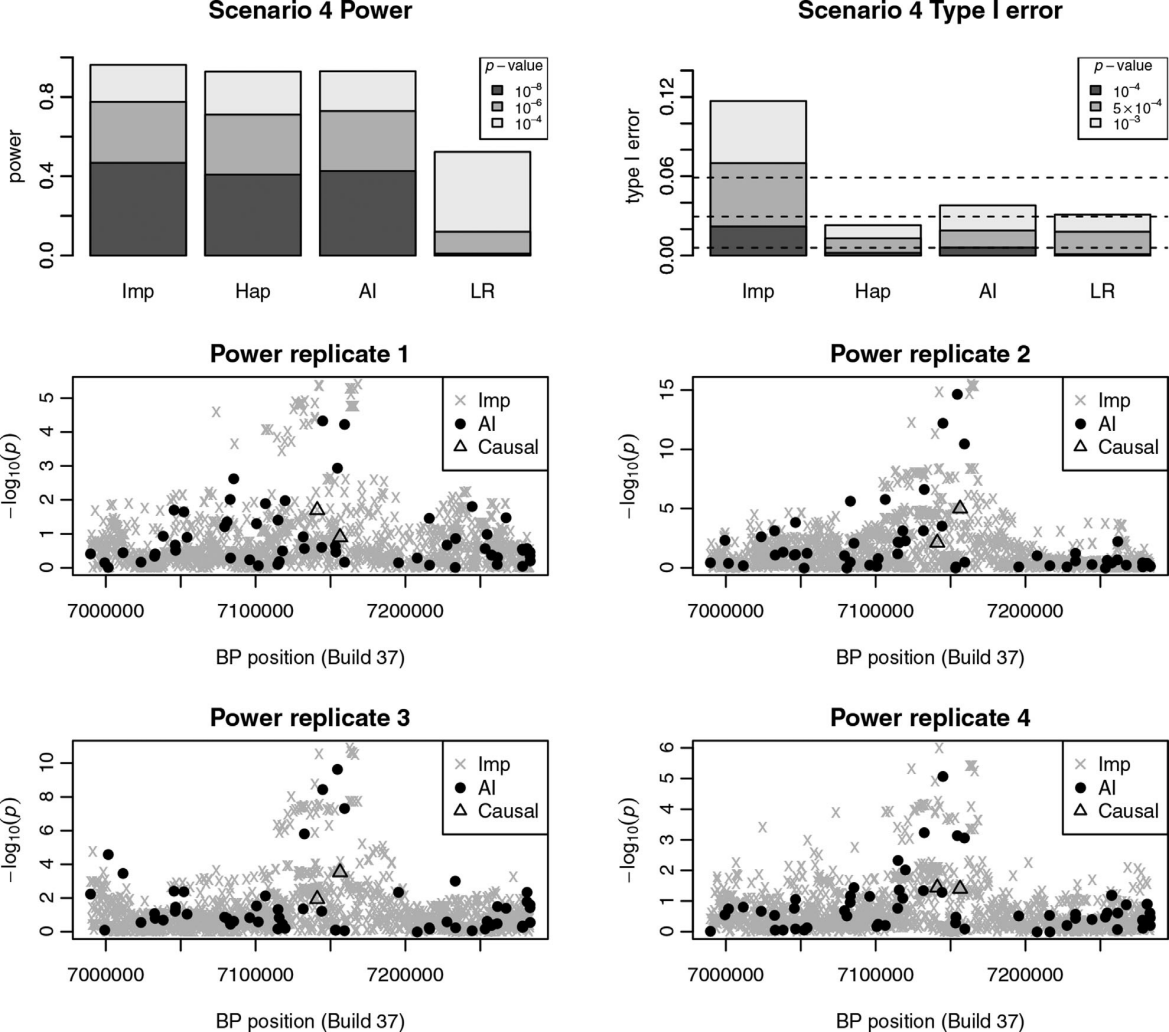
Results from Scenario 4 (haplotype effects). The top plots shows bar plots of the calculated powers (for *P*-values 10^−8^, 10^−6^, and 10^−4^) and type I errors (for *P*-values 10^−4^, 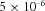, and 10^−3^) for imputation (Imp), haplotype analysis (Hap), single-SNP logistic regression (LR), and the AI test. The bottom plots show examples of results from four separate power replicates. Gray crosses show the results obtained from imputation, black dots show the results obtained from AI, and triangles show the imputation results obtained at the two causal SNPs. The default window size of 10 SNPs was used in the AI test.

Figure 3 (top) shows the detection power and type I error for standard imputation, haplotype analysis, AI, and single-SNP logistic regression for data simulated under a more complicated scenario (Scenario 4), a haplotype model in which disease was assumed to be caused by a haplotype effect defined by two underlying causal nongenotyped SNPs, rs7926004, and rs10500679. Possession of a T-G haplotype at these two SNPs was assumed to increase the risk of disease by a factor of 1.8, while possession of a C-C haplotype at these two SNPs increased the risk of disease by a factor of 1.5, in comparison to haplotypes T-C and C-G. As in Scenarios 1-3, standard imputation, haplotype analysis and AI considerably outperform single-SNP logistic regression in terms of detection power, although the type I error is greater for imputation than for AI or haplotype analysis. We were not surprised to find that haplotype analysis performed well in this scenario, even though the causal SNPs were not genotyped. This result is loosely consistent with the results of Morris and Kaplan [2002], who showed that haplotype analysis performed better than single SNP testing when disease was attributable to multiple alleles at a single locus; from a statistical point of view, a haplotype effect at two unobserved loci could be considered statistically equivalent to multiple susceptibility alleles at a single unobserved locus. We were surprised that standard imputation showed such high power, as this scenario was specifically designed to encapsulate a situation where there is a strong haplotype effect that results in much weaker marginal effects at each of the contributing SNPs, when analyzed individually. The bottom plots of Figure 3 go some way toward explaining this slightly counter-intuitive result. In each of these example replicates, we see that both AI and standard imputation are able to capture a signal in the vicinity of the two causal SNPs. However, the signal captured by imputation is not, in fact, a signal at either of the causal SNPs, which have been well imputed but show only weak marginal effects, as expected. Rather, the imputation signal comes from other well-imputed SNPs in the region that presumably mark the causal haplotype. Just as haplotype analysis can capture the effect of an untyped causal variant through testing a haplotype that marks (i.e., is a good surrogate for) the untyped causal variant, it seems that imputation can capture the effect of an underlying causal *haplotype* through testing a SNP that marks this underlying causal haplotype. This is a potentially attractive property of imputation that has not, to our knowledge, been previously demonstrated. However, it does raise an important issue regarding the interpretation of imputation results. Given a set of imputation results such as those seen in the bottom plots of Figure 3, the usual interpretation would be that the signal is due to a causal effect at the top scoring SNP (or possibly due to a causal effect at another SNP in strong LD with the top scoring SNP). Our results demonstrate that this is by no means the only explanation for such a signal. Indeed, our results suggest that it is not possible, statistically speaking, to distinguish between this simple explanation and other, more complicated explanations; distinguishing between different possible explanations may require different types of experiment, based on different types of data.

**Figure 4:**
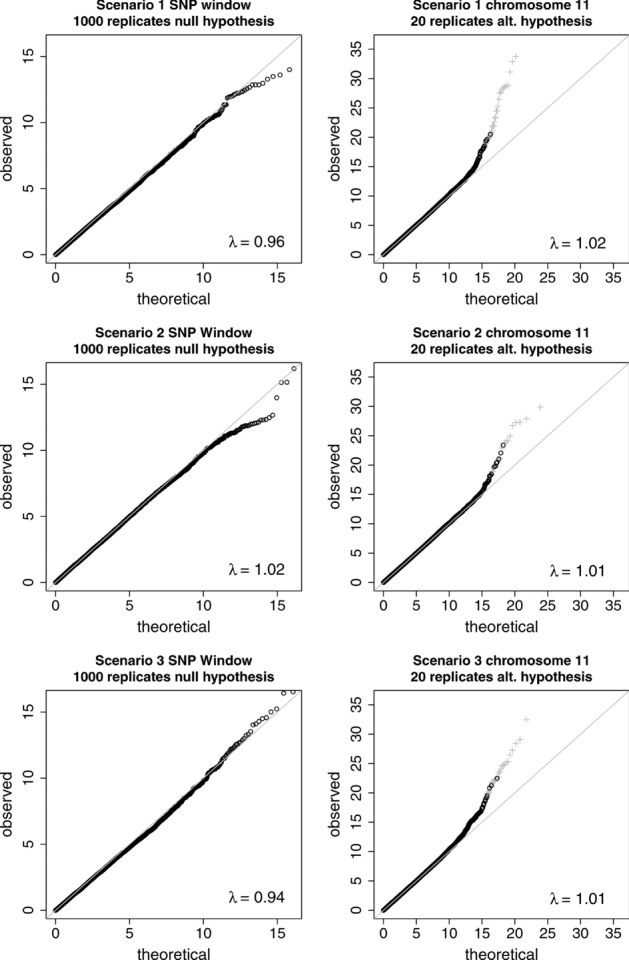
Q-Q plots of the AI test statistics for Scenarios 1–3. The plots on the left show results from 1,000 replicates under the null hypothesis with no causal variants. The plots on the right show results from 20 replicates of the whole of chromosome 11 under the alternative hypothesis, where crosses denote SNPs that are within 110 K base pair positions of the causal SNP.

As an additional check on the type I error rate for AI, we constructed quantile-quantile (Q-Q) plots and calculated genomic control inflation factors [Devlin and Roeder, 1999], λ, for the AI test statistics from Scenarios 1-3. Figure 4 (left hand plots) shows the test statistics obtained within the 110 kb detection window for 1,000 replicates simulated under the null hypothesis. Figure 4 (right hand plots) shows the results for 20 replicates of the whole of chromosome 11 simulated under the alternative hypothesis, where gray crosses indicate test statistics of SNPs that lie within the 110 kb detection window, which might therefore be considered as “true” findings. The Q-Q plots of the test statistics show an acceptable distribution, indicating that the AI approach provides inference that can be considered to have essentially the same properties as inference from standard single-SNP testing in GWAS.

The effect of using a dominant or recessive analysis model and/or a dominant or recessive correlation metric, when the causal SNP was simulated to operate under a dominant or recessive model, was also investigated for Scenarios 1-3. Supplementary Figure S3 shows the powers for single-SNP logistic regression and the AI test using a SNP window size of 10 SNPs. Single-SNP logistic regression using the correct analysis model performed worse than using a multiplicative analysis model, as previously demonstrated [Iles, 2010] for SNPs in low LD with a causal SNP. This observation extends to the AI test: when a dominant/recessive regression model is used (see Methods) the power is lower than when using a multiplicative model, even for data simulated under a dominant/recessive model. Our implementation of the AI test therefore always uses a multiplicative analysis model. Using a *correlation metric* constructed for a dominant/recessive causal SNP (see Methods) when the causal SNP really is dominant/recessive tends to perform slightly better than using one constructed for a multiplicative causal SNP. However, the difference in power is not large and the multiplicative metric still has higher power in some instances. For AI we therefore recommend using the multiplicative correlation metric unless there is a strong prior belief in a dominant or recessive causal variant.

### Application to MalariaGEN Case/Control Data

As a demonstration of our method applied to real data, we used a case/control study of severe malaria from The Gambia, genotyped on the Affymetrix 500K array (data provided by the MalariaGEN consortium, www.MalariaGEN.net). It is expected that many SNPs may be poorly tagged as this array was originally developed for European rather than African populations. The original dataset consisted of 1,059 cases and 1,496 controls typed at around 500,000 SNPs. These data were previously analyzed by Jallow et al. [2009], who performed quality control resulting in the retention of 958 cases, 1,382 controls, and 402,814 SNPs, where, in addition to automated SNP exclusions, many poorly-clustering SNPs (with respect to cluster plots of the intensity values) were removed “by eye.” Unfortunately we were unable to obtain precise lists of which cases, controls and SNPs had been removed, so we performed our own (slightly more stringent) quality control within the computer package PLINK [Purcell et al., 2007], resulting in the retention of 831 cases, 1,287 controls, and 328,399 SNPs (with no SNPs removed by eye). We focus here initially on the results from chromosome 11, which consisted of 17,515 SNPs.

**Figure 5:**
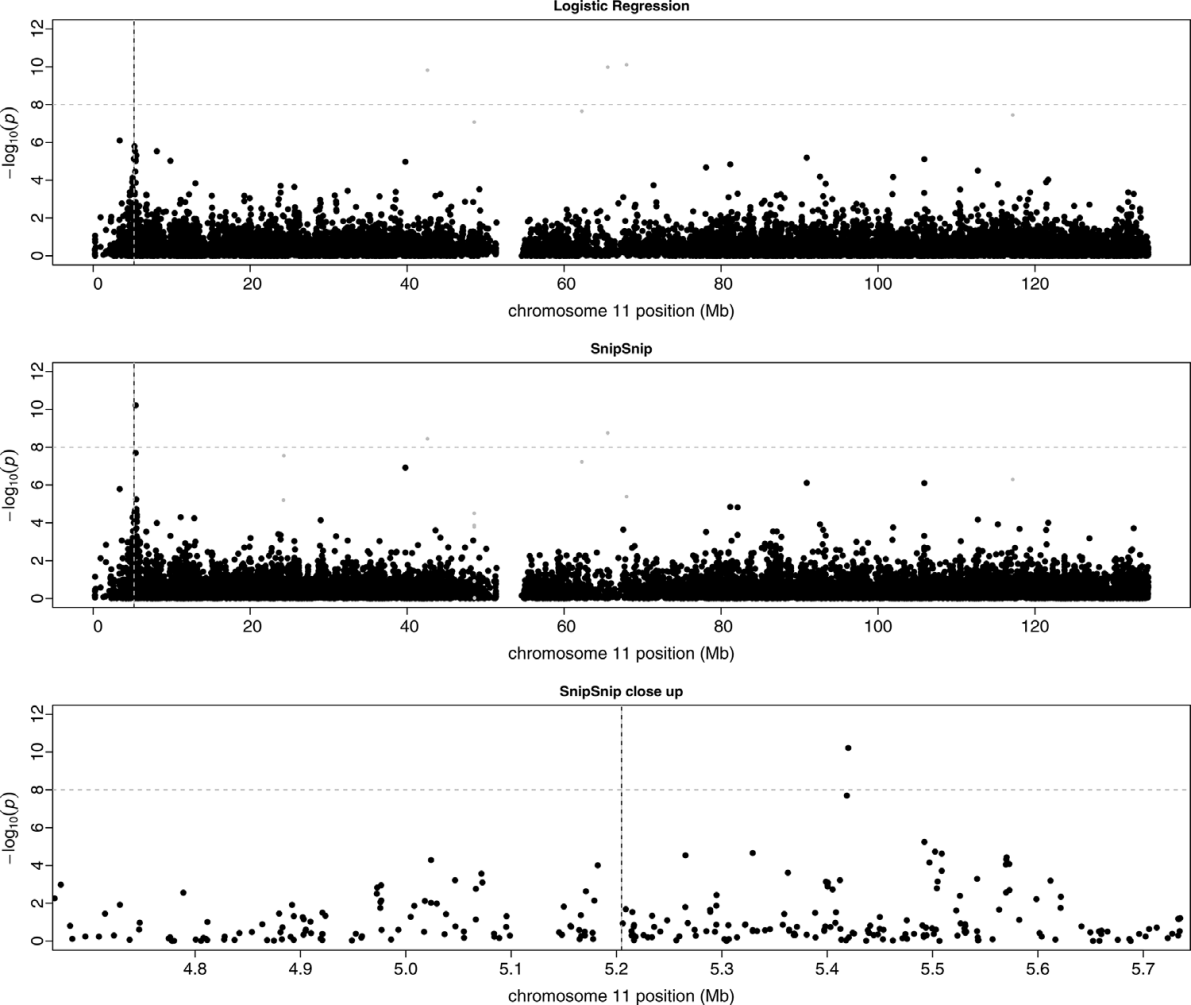
Manhattan plots on chromosome 11 for the case/control severe malaria dataset from The Gambia. The top plot shows single-SNP logistic regression *P*-values obtained from analysis in PLINK. The middle plot shows AI test *P*-values. The lower plot shows a close-up of the AI test *P*-values around the causal SNP. The horizontal dashed line shows 10^−8^ (a common threshold indicating genome-wide significance). The vertical dashed line indicates the position of the causal SNP, rs334. Light gray points show SNPs with logistic regression *P*-value below a threshold of 10^−7^, which were presumably removed (by eye, following cluster plot checks) in the analysis by Jallow et al. [Jallow et al., 2009]

Figure 5 (top) shows a Manhattan plot of the 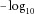 *P*-values for chromosome 11 in the Gambian case/control dataset using single-SNP logistic regression implemented in PLINK. To adjust for population stratification, we first performed principal component analysis (PCA) using the *smartpca* routine of the EIGENSOFT package [Price et al., 2006] on 139,445 autosomal SNPs (pruned to be in low levels of LD with one another using the PLINK command “–indep 50 5 2”). The first three principal components from *smartpca* were then included as explanatory variables in the logistic regression models fitted by PLINK. (Note that Jallow et al. [2009] also included the first three principal components to correct for population stratification, within a standard logistic regression framework). Colored in light gray in Figure 5 (top) are points that appear to be spurious associations and do not appear in the corresponding Manhattan plot shown in Jallow et al. [2009]; we presume that these correspond to untrustworthy poorly-clustering SNPs that were removed “by eye.” A weak association signal (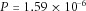 at rs11036635) can be seen in the vicinity of the known causal SNP, rs334, whose position is shown as a dashed vertical line.

Figure 5 (middle) shows the results obtained from AI analysis in SnipSnip (with the first three principal components included as covariates and the same presumed untrustworthy SNPs colored in gray). The most significant SNP (rs16931041) has a *P*-value of 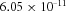 and lies 215 kb from the known causal SNP, rs334. The lower plot shows a close-up plot of the AI results in this region. The AI results are considerably more compelling than the results from standard logistic regression in the region of this known causal SNP, comfortably reaching genome-wide levels of significance. The overall genomic control inflation factor (from all 328,399 SNPs tested across the genome) was 1.07 for the AI test, just within acceptable levels for a GWAS. Without adjustment for population stratification the genomic control inflation factor was 1.21. Standard logistic regression in PLINK gave genomic control inflation factors of 1.08 (with adjustment for population stratification) or 1.29 (without adjustment for population stratification), respectively.

To examine the source of the association signal identified by SnipSnip, we obtained from the MalariaGEN investigators genotype data (generated using the Sequenom iPlex platform) for the Gambian case/control samples at the known causal SNP, rs334. Table1 shows estimated haplotype frequencies and expected haplotype counts (estimated separately within cases and controls) for haplotypes consisting of the disease, partner, and anchor SNPs, generated using the software UNPHASED [Dudbridge, 2008; Dudbridge et al., 2011]. Allele A at rs334 is protective, having a much higher frequency in controls than cases. This protective allele occurs exclusively in coupling with either a C-C or C-T haplotype at the partner and anchor SNP respectively, although the nonprotective T allele at rs334 additionally occurs in coupling with T-C (or very occasionally with T-T) haplotypes. Logistic regression analysis using either expected haplotype counts or observed genotype variables, with three principal components again included as covariates, indicated that, once rs334 was included in the model, neither the anchor SNP (rs16931041), the partner SNP (rs2340349), nor the test of the anchor SNP given the partner SNP were significant (all 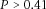) suggesting that the significance seen in the original AI test could indeed be accounted for by genotype at the true causal SNP, rs334. Haplotype analysis of the partner and anchor SNPs alone using UNPHASED [Dudbridge, 2008; Dudbridge et al., 2011] (cases and controls combined, including the first three principal components as covariates) indicated that haplotypes at these SNPs were significantly associated with disease status at stronger significance levels (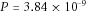) than either the partner (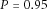) or anchor (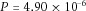) SNP alone; the significance of adding the anchor SNP into a haplotype model that already includes the partner SNP was 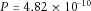, highly consistent with the results from the similar test implemented in SnipSnip.

**Table 1:** Estimated haplotype frequencies and expected counts for haplotypes consisting of the causal SNP (rs334), partner SNP (rs2340349) and anchor SNP (rs16931041) in MalariaGEN case/control data

Haplotype (rs334-partner-anchor)	Case frequency	Control frequency	Case count	Control count
A-C-C	0.005611	0.053530	8.978	131.700
A-C-T	0.001889	0.021680	3.022	53.330
A-T-C	0.000000	0.000000	0.000	0.000
A-T-T	0.000000	0.000000	0.000	0.000
T-C-C	0.026310	0.026960	42.100	66.330
T-C-T	0.922400	0.853100	1,476.000	2,099.000
T-T-C	0.043080	0.044720	68.930	110.000
T-T-T	0.000671	0.000000	1.074	0.000

Table2 shows correlations (assuming an additive allelic coding) between rs334 and the top anchor SNP (rs16931041), between rs334 and the top partner SNP (rs2340349) and between rs334 and a “combination” variable constructed by adding together the genotype variables at rs16931041 and rs2340349. The anchor/partner combination variable is seen to be considerably more correlated with rs334 than are either the anchor or partner SNP alone. Although this observation does not precisely correspond to the AI test performed in SnipSnip, there is a loose correspondence between these results. In particular, this observation illustrates that the information provided by the anchor and partner together (each coded in an allelic fashion) is a reasonably good surrogate for the information that would be provided by rs334 alone, explaining why a logistic regression model that includes both rs16931041 and rs2340349 as predictors predicts disease status better than either variable individually, and why such a model provides a reasonable approximation to the “gold-standard” model that would include, instead, a single predictor consisting of genotype at the true causal variant.

**Table 2:** Squared correlation coefficients (*r*^2^) between the causal SNP (rs334) and the anchor SNP (rs16931041), partner SNP (rs2340349) or additive anchor/partner SNP combination, in MalariaGEN case/control data

	*r*^2^ based on haplotypes			*r*^2^ based on genotypes		
SNP pair	Controls	Cases	Combined	Controls	Cases	Combined
rs334-partner	0.004	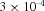	0.002	0.002	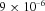	0.001
rs334-anchor	0.255	0.043	0.199	0.258	0.055	0.205
rs334-anchor/partner combination	0.438	0.109	0.374	0.446	0.149	0.397

### Application to MalariaGEN Trio Data

We also analyzed an additional MalariaGEN Gambian case-parent trio dataset, consisting of 658 severe malaria cases and their parents genotyped on the Illumina 650Y array (584,945 SNPs). Our method is designed for analysis of case/control rather than trio data, but trio data can be analyzed by converting it to cases and pseudocontrols (with pseudocontrols constructed based on the alleles not transmitted from the parents to the case, see Cordell and Clayton [2002] and Cordell et al. [2004] for details). The resulting case/pseudocontrol dataset was then analyzed using both our approach and standard single-SNP logistic regression, and compared with a trio analysis using the transmission disequlibrium test (TDT) [Spielman et al., 1993] implemented in PLINK.

**Figure 6:**
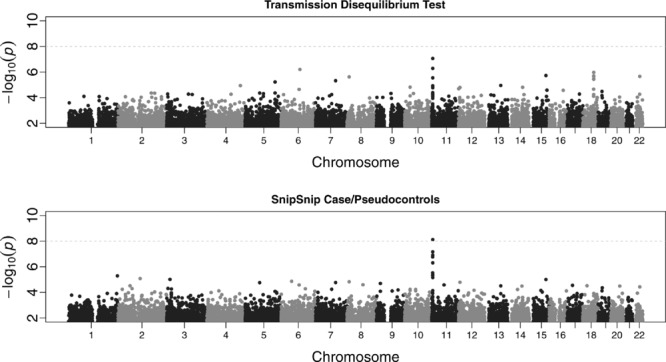
Manhattan plots of each chromosome for the case-parent trio severe malaria dataset from The Gambia. The top plot shows *P*-values using the transmission disequilibrium test as implemented in PLINK. The lower plot shows AI test *P*-values. The black and gray points highlight different chromosomes.

Figure 6 shows Manhattan plots across the whole genome for the Gambian case-parent trio dataset, using either the TDT implemented in PLINK (top panel) or the AI test implemented in SnipSnip applied to cases and pseudocontrols (bottom panel). The overall genomic control inflation factor was 1.02 for the TDT and 1.00 for the AI test. Both methods gave reasonably compelling results in the vicinity of the known causal SNP (rs334) on chromosome 11, but the top AI result (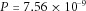 at rs979752) was an order of magnitude more significant than the top TDT result (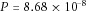). Standard logistic regression applied to same case/pseudocontrol data gave similar results to the TDT but with less significant *P*-values. The top anchor (and partner) SNP in the case-parent trio study differed from that found in the case/control study, most likely due to the different genotyping arrays used in the two studies, which contain SNPs that show varying patterns of LD with the causal SNP, rs334.

### Imputation Analysis of MalariaGEN Data in rs334 Region

**Figure 7:**
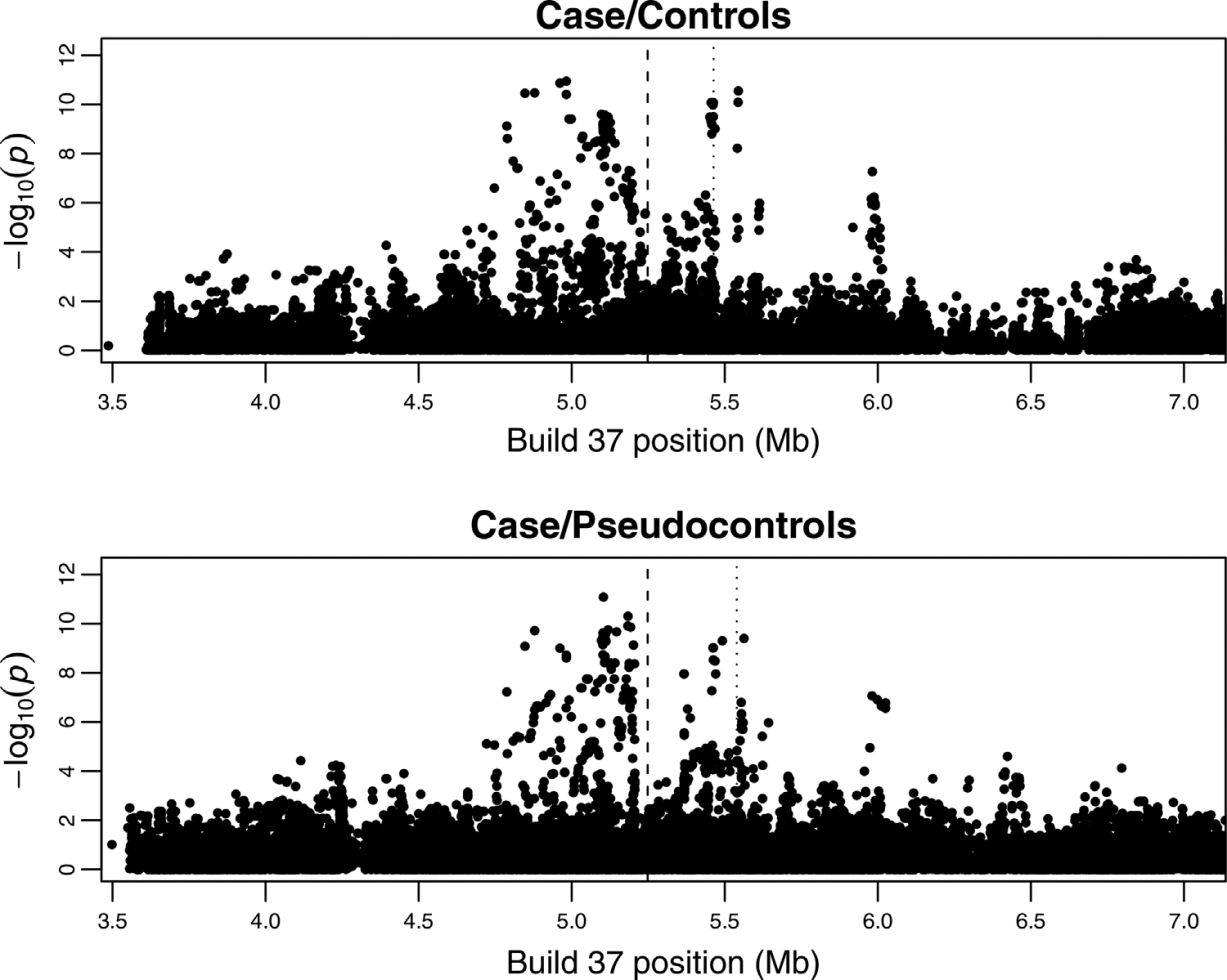
Imputation results for Gambian case/control and trio (case/pseudocontrol) datasets. The position of the causal SNP, rs334 (not present in 1000 Genomes) is shown with a vertical dashed line. The position of the top anchor SNP identified by SnipSnip is shown with a vertical dotted line.

We carried out imputation (without prephasing) within the Gambian case/control samples, and within the cases and pseudocontrols derived from the Gambian case-parent trio samples, in the 4 Mb region around the known causal SNP (rs334) on chromosome 11. We used the program IMPUTE2 [Howie et al., 2009; Marchini et al., 2007] with data from the 1000 Genomes Project [1000 Genomes Project Consortium et al., 2012] (Phase I interim data, updated release April 2012) as a reference panel. 22,907 SNPs (in the case/control data) or 31,757 SNPs (in the case/pseudocontrol data) passing postimputation quality control (“info” score >0.5) from an original 66,754 imputed SNPs were analyzed using the “-method threshold” method in the program SNPTEST (allowing for the first three principal components as covariates in the case/control analysis) to test for association with disease status. Figure 7 shows the results from this analysis. Imputation followed by single-SNP analysis is able to improve the signal of association to 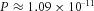 in the case/control dataset (compared to 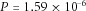 seen previously when using real genotyped SNPs alone) and to 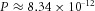 in the trio dataset (compared to 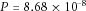 seen previously when using real genotyped SNPs alone). Thus, the signals detected through the AI test in SnipSnip could potentially have been detected through use of genome-wide imputation. However, imputation on a genome-wide scale is computationally demanding and requires careful postimputation quality control and filtering to remove untrustworthy results. An initial (much faster) analysis using SnipSnip could allow one to focus one's imputation efforts on the most promising regions before embarking on a full genome-wide imputation analysis. In the current example, imputation of this 4 Mb region (comprising 960 genotyped SNPs) in the 2,118 Gambian case/control individuals using IMPUTE2, followed by single-SNP analysis in SNPTEST, took approximately 18 h on our Linux system (not including the time required to reformat files, including performing a liftover from Build 36 to Build 37 positions in order to match up the study samples with the 1000 Genomes data); we estimate that to carry out the same analysis across the entire genome (328,399 genotyped SNPs) would have taken over 36 weeks. This time can, of course, be reduced substantially through pre-phasing [Howie et al., 2012] and/or implementation in parallel on a compute cluster (see whole-genome imputation results presented below), but still compares unfavorably with the 37 min (with covariates) or 44 sec (without covariates) taken by SnipSnip (which can also similarly be reduced through implementation in parallel) to perform a whole-genome analysis on the same samples.

We note that the signal at rs334 detected here using 1000 Genomes Project samples as a reference panel for imputation (Fig. 7) is considerably weaker than the imputed signal (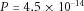) found at rs334 by Jallow et al. [2009] when using an arguably more appropriate (but not publicly available) reference panel of 62 Gambian controls that they resequenced themselves in the rs334 region. To our knowledge, Phase I of 1000 Genomes Project does not contain any Gambian samples (although their inclusion is planned for later releases), nor did the reference panels we used (provided on the IMPUTE2 website) contain the actual causal SNP, rs334. Nevertheless, it seems that the effect of rs334 can be at least partly detected via its LD pattern with SNPs that have been reliably called within populations currently included in 1000 Genomes. Interestingly, our imputation results around rs334 using 1000 Genomes as a reference panel do show considerably stronger signals of association than were obtained by Band et al. [2013] on a partially-overlapping set of Gambian samples using HapMap3 as a reference panel, possibly due to the better coverage of SNPs marking rs334 provided by the 1000 Genomes Project compared to HapMap3. This observation illustrates the importance of (and potential sensitivity to) the choice of reference panel when conducting imputation.

### Genome-Wide Imputation Analysis of MalariaGEN Data

We additionally carried out genome-wide imputation with prephasing [Howie et al., 2012] using the programs IMPUTE2 [Howie et al., 2009; Marchini et al., 2007] and SHAPEIT [Delaneau et al., 2012]. 263,565 genotyped SNPs (case/control data) or 561,510 genotyped SNPs (trio data) passing pre-imputation quality control (minor allele frequency >0.01, 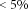 missing genotypes, no A/T or C/G SNPs or other possible strand discrepancies) were used to generate tests (using the “-method threshold” method in the program SNPTEST) at up to 13.2 million imputed SNPs that passed post-imputation quality control (“info” score >0.5 and minor allele frequency >0.01). Supplementary Figure S4 shows the Manhattan plots obtained, together with results from AI. Imputation results on odd-numbered chromosomes are colored in pink and those on even-numbered chromosomes are colored in green; black circles represent the AI results. For the case/control dataset (upper plot), the results are dominated by a signal at rs12315364 on chromosome 12, which is detected by both AI and standard imputation. Given the extreme significance of this result, the fact that it was not reported by Jallow et al. [2009] and the fact that it does not appear in the lower (case/pseudocontrol) Manhattan plot, it seems likely that this is an artifactual finding caused by poor-quality genotype data at rs12315364. This conclusion is substantiated by the fact that the signal completely disappears when the imputation exercise is repeated with rs12315364 removed from the set of genotyped SNPs used to inform imputation (middle plot). A number of other (presumed artifactual) signals remain; we assume that these correspond to untrustworthy poorly clustering SNPs that were removed (postanalysis) by Jallow et al. [2009]. With respect to rs12315364, it is interesting that inclusion of a single poor-quality SNP can result in such a large number of imputed SNPs (which pass post-imputation quality control) apparently marking the same signal; we have noticed this phenomenon occurring in other imputed datasets that we have analyzed. In a standard GWAS (of genotyped SNPs only), the presence of a number of SNPs in the same region showing the same signal is generally considered an indication that the signal is reliable; however, the same can clearly not be said for an imputation-based GWAS. Although such artifacts would hopefully be picked up through careful inspection of all interesting signals (including inspection of cluster plots for those genotyped SNPs in the region), it does highlight the importance of carrying out careful quality control of the real genotype data before embarking on imputation. In this instance, it is unclear whether the problem with rs12315364 would have been automatically detected becaus it is not, in fact, significant (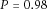) when analyzed in PLINK using single-SNP logistic regression (with 3 principal components included as covariates), although it is highly (and suspiciously) significant (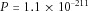) when analyzed instead using the Armitage trend test.

For the case/control dataset, the results shown in supplementary Figure S4 at the known causal SNP, rs334 on chromosome 11, are scarcely visible on the background of presumed artifactual signals, but for the trio (case/pseudocontrol) dataset, the signal at rs334 is much clearer. Both imputation and AI are successful in detecting the location of the true causal SNP, with imputation achieving an overall higher level of statistical significance. However, the clearly visible imputation results (pink and green crosses) seen poking above the AI results (black dots) across the whole genome illustrate the higher number of false-positives expected from genome-wide imputation analysis, if the significance threshold for “detection” is set at standard genome-wide significance levels, consistent with our simulation results presented earlier.

It took about 10 days to carry out genome-wide pre-phasing and imputation of these two datasets, which was enabled by running the analyses for each of the 22 chromosomes in parallel; without access to a compute cluster we anticipate this analysis would have taken several months. More importantly, prephasing and imputation (followed by analysis in SNPTEST) of these two datasets generated around 270 GB (for the case/control dataset) and 488 GB (for the case/pseudocontrol dataset) of output and intermediate files respectively, i.e., a total of 758 GB of storage was required in order to run this experiment. In comparison, analysis using AI in SnipSnip took between 1 second and 2.5 min per chromosome and generated a mere 105 MB of output files.

### Genome-Wide Haplotype Analysis of MalariaGEN Data

We also carried out genome-wide haplotype analysis of the MalariaGEN data in UNPHASED, using a sliding window of 5-SNP haplotypes (see supplementary Fig. S5). For the case/control dataset (top plots), similar to supplementary Figure S4, the signal at the known causal SNP rs334 is scarcely visible on the background of presumed artifactual signals, regardless of whether or not “suspicious” SNP rs12315364 is excluded. For the case/pseudocontrol dataset (bottom plot), the signal at rs334 is clearly visible, reaching similar significance to that seen using imputation. In theory, greater power for haplotype analysis could be obtained by keeping trios intact rather than analyzing them as cases and pseudocontrols. However, we found that the default maximization procedure in UNPHASED did not converge reliably with the trio dataset, generating large numbers of presumed erroneous artifactual signals. We found this problem in UNPHASED could be avoided by use of the slower (Nelder-Mead) maximization option, however, this option was not feasible on a genome-wide scale; we estimated that the analysis would have taken several months to run on our system, even when divided into 22 (or more) parallel jobs. Analysis of the case/pseudocontrol dataset in UNPHASED (using the default maximization option) was relatively fast, taking around 12 h to process the longest chromosomes and producing a total of 875 MB of output files. Analysis of the case/control dataset was much slower on account of including three principal components as covariates; it took around 10.5 days to process the longest chromosomes and produced a total of 665 MB of output files.

## Discussion

Here we have described a new method and accompanying software package, SnipSnip, that is designed for analysis of GWAS data to improve the signal of association in situations where there are a number of SNPs in low LD with the causal variant. Our computer simulations demonstrate that, in some circumstances (depending on the LD structure between the causal variant and SNPs that have been genotyped, within the population under study), we can gain a considerable boost in power compared to single-SNP analysis. Our application to the MalariaGEN data illustrates the utility of our approach in identifying, at genome-wide levels of significance, a region known to harbor a causal SNP that confers protection from malaria.

One might expect that, in many cases, imputation analysis using an appropriately chosen reference panel could reproduce the signals identified by SnipSnip. Indeed, our goal in developing AI was not to generate a method that necessarily showed any *higher* power than imputation, but rather to generate a method that captured *much of the signal* that is captured by imputation, while taking a fraction of the time, computational effort and disc space. Both AI and imputation capitalize on the fact that, although there may be no genotyped SNPs in strong LD with the causal variant, there may be several genotyped SNPs in weak LD with the causal variant that, when considered together, provide equivalent information. However, capturing this signal through genome-wide imputation is computationally demanding, requires careful postimputation filtering and quality control to produce reliable results, and generally requires a relatively high level of expertise in scripting (e.g., shell scripts, Perl scripts, Python scripts etc.) as well as access to (and familiarity with using) high performance computing facilities such as a multinode Linux cluster, if the analysis is to be performed in a reasonable time. Setting up the required input files and pipelines for analysis (including performing a “liftover” to the current genome build and checking strand alignments) can be quite fiddly and, in our experience, even well-qualified and experienced researchers can make inadvertant mistakes when setting up these analyses, frequently resulting in an analysis, or part of an analysis, needing to be repeated. Finally, the output (and intermediate) files generated by this procedure are often unwieldy, taking up many GB of disc space. Clearly none of these concerns are major obstacles for the numerous well-funded and appropriately staffed research groups and consortia that routinely use imputation, but they do highlight the attraction of a convenient (and much faster) complementary approach such as the AI test proposed here. The question is not, perhaps, so much, whether AI improves over imputation, but whether imputation analysis improves significantly over AI, given its higher computational (including disc space) and manpower cost.

On that note, standard imputation is obviously a much more attractive option if the goal is to generate common panels of SNPs (genotyped or imputed), in order to enable large-scale meta-analyses of studies that have been carried out using different genotyping arrays [Band et al., 2013; Berndt et al., 2013; Zeggini et al., 2008]. Similarly, if one genuinely wishes to infer (albeit probablistically) nongenotyped SNPs within a set of study samples (perhaps as a precursor to performing some kind of rare-variant analysis [Mägi et al., 2012]), imputation is again an extremely convenient approach. Our quarrel is not with imputation per se, which we consider an elegant and effective analysis tool, but rather with the fact that imputation is often presented as itself being the main goal of an analysis, whereas for many researchers the main (and more important goal) is relating genotypic variation to phenotypic variation. For that purpose, at least when surveying common variation in a single study dataset from a population that is well-tagged by current genotyping arrays, it is unclear whether either AI or imputation offers any great advantage over single-SNP association testing. However, for populations that are less well-tagged by the array that has been used, both AI and imputation could be used to improve the signal of association at poorly tagged causal variants, with AI performing the analysis in a fraction of the time.

A drawback of standard imputation is that it, in principal, requires access to an appropriate reference sample; results can be highly sensitive to the reference panel used (as seen from the disparity between our imputation results around the MalariaGEN causal SNP, rs334, using 1000 Genomes as a reference panel, compared to those obtained by Band et al. [2013] using HapMap3 as a reference panel). For populations that are not closely related to populations for which dense SNP data or genome-wide sequence data is currently available, this can be a problem. The AI test implemented in SnipSnip does not require access to an external reference panel, making use only of the LD pattern amongst SNPs genotyped within the study samples (together with their multimarker correlation with a disease or quantitative phenotype) to construct a test of association. This fact means that there may even be situations in which the AI test implemented in SnipSnip succeeds better than imputation. One such situation is if the causal variant has only recently arisen within the population under study, and does not appear in (or show a strong LD pattern with SNPs genotyped in) *any* available reference panel. The extent to which this will occur in practice is unknown, but it is not inconceivable that there may be situations where this occurs, particularly, for example, in studies of indigenous or isolated populations [Bourgain et al., 2001; Gusev et al., 2011; Zhang et al., 2013].

Another situation where one might, in principal, expect AI to succeed better than imputation is when the causal association is due to a genuine haplotype effect (perhaps caused by interactions between nearby SNPs) and not to the effect of any single untyped SNP. Our simulation results from Scenario 4 suggest that this type of signal may, in fact, be detectable through standard imputation, although this comes with a higher cost in terms of the probability of making a type I error. Although methods for genome-wide haplotype analysis exist [Allen and Satten, 2009a, 2009b; Browning and Browning, 2007a, 2008; Gusev et al., 2011], these approaches, like imputation, tend to be highly computer-intensive and have not been frequently used, possibly because of this fact as well as their requirement for extensive postanalysis quality control to produce reliable results [Browning and Browning, 2008]. Prior to the GWAS era, haplotype methods (or at least the development thereof) achieved high popularity [Bourgain et al., 2002; Chapman et al., 2003; Clayton and Jones, 1999; Clayton, 1999; Cordell, 2006; Dudbridge et al., 2000; Durrant et al., 2004; Lin et al., 2004; Molitor et al., 2003; Morris, 2005, 2006; Schaid et al., 2002; Seltman et al., 2001; Stram et al., 2003; Templeton et al., 1987, 1988, 1992; Templeton and Sing, 1993; Toivonen et al., 2000; Tzeng et al., 2003; Zaykin et al., 2002] but, in practice, since the advent of GWAS, such tests have rarely been applied on a genome-wide scale, perhaps on account of their computational burden combined with a perception that such methods have been superceded by imputation.

Our method has some similarities with previously proposed multimarker tests that either directly test sets of adjacent (or nearby) SNPs [Chapman et al., 2003; Clayton et al., 2004; Humphreys and Iles, 2005; Kim et al., 2010; Slavin et al., 2011; Wason and Dudbridge, 2010] or else that construct tests at untyped SNPs on the basis of the multimarker genotypes observed [Allen et al., 2010; de Bakker et al., 2005; Lin et al., 2008; Nicolae, 2006; Pe'er et al., 2006]. A full comparison of our approach with these previously proposed methods is beyond the scope of the current manuscript, but would be an interesting topic for further investigation. Approaches that rely on observed multimarker genotypes to construct tests at untyped SNPs have a close relationship to imputation-based approaches, relying on a reference panel (such as HapMap or 1000 Genomes) to infer information at untyped SNPs on the basis of their correlation pattern (in the reference panel) with genotyped SNPs. These approaches can be computationally intensive although some faster implementations do exist [Allen et al., 2010]. Approaches that directly test sets of adjacent (or nearby) SNPs, have been shown to improve power over single-SNP testing in simulation studies [Kim et al., 2010; Wason and Dudbridge, 2010], although it is less clear whether this power improvement is always achieved in practice: Slavin et al. [2011] had some success when applying this approach to GWAS data for coronary artery disease and hypertension, while Wason and Dudbridge [2010] had less success applying it to schizophrenia. A problem with the simultaneous testing of several adjacent SNPs is the increased degrees of freedom (df) incurred; the main-effects test used by Wason and Dudbridge [2010] and Slavin et al. [2011] has 2 df while their main+adj test has 3 df, in contrast to the 1 df AI test proposed here. The main-effects test corresponds essentially to comparing our model 1 with the global null hypothesis that neither SNP is associated with disease status (i.e., 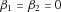), while the main+adj test corresponds to adding in an anchor-partner interaction term and comparing the resulting 3 df model with the global null hypothesis that neither SNP (nor the interaction) is associated with disease status. Part of the motivation for developing AI was our intuition that, in a scenario where the 2 df main-effects test is more powerful than than the single-SNP test, then model 1 with two main effects should, by definition, fit these data better than model 2 with one main effect (at the partner SNP) only. In AI, we assess this directly through use of a 1 df (conditional) test. Another difference between our approach and previously proposed approaches is the fact that we have developed a specific algorithm to choose the “optimal” partner SNP for each tested anchor SNP. In contrast, Wason and Dudbridge [2010] and Slavin et al. [2011] use pairs of adjacent SNPs (possibly subject to an “LD pruning” step to filter out SNPs that are almost completely inferrable from nearby SNPs). It is not obvious that pairs of adjacent SNPs will necessarily be the optimal unit for this type of analysis; in construction of our correlation metric we found the correlation between the SNPs to be a useful measure of their compatibility, at least with respect to the AI test.

Our proposed method makes use of conditional regression models (testing the effect of one predictor *conditional* on the inclusion of another in the regression model). Such conditional regression models are not new in the genetics literature; following a suggestion by Cordell and Clayton [2002], conditional or stepwise regression strategies have commonly been used to help select independent variants and/or model multiple loci that are in LD in a variety of different studies [Barratt et al., 2004; Broadbent et al., 2008; Cordell et al., 2013; Fellay et al., 2007; Haiman et al., 2007; Hunt et al., 2013; Knight et al., 2012; Lincoln et al., 2005; Liu et al., 2012; Plenge et al., 2007; Scott et al., 2007; Trynka et al., 2011; Ueda et al., 2003]. However, the use of such conditional regression models has generally been confined to dissecting or accounting for signals that have *already* been detected through single-SNP testing; to our knowledge such strategies have not generally been used within sliding windows on a genome-wide scale, with a view to detecting *new* signals at additional genomic locations (over and above those locations detected through single-SNP testing), as proposed here.

In conclusion, we propose here a complementary test to more complex methods (such as sequencing and imputation or haplotype analysis) that takes only a fraction of the time/computational complexity/disc space of these more complex methods, and may be used with standard GWAS data, without any requirement for additional reference samples, to identify genetic regions of interest. Software implementing our method is freely available from http://www.staff.ncl.ac.uk/richard.howey/snipsnip/.

## Supporting Information

Disclaimer: Supplementary materials have been peer-reviewed but not copyedited.

**Figure S1**. Plots of the correlation metrics used in the AItest to choose a partner SNP. The solid black line shows the fitted curve which maps the anchor-partner SNP correlation to a score in the interval [0, 100]. The black dots show the evaluated correlation values from simulations. Both the dots and fitted curve have been rescaled so the peak of the curve is at value 100. From left to right the correlation metrics are defined for causal variants that are: multiplicative, dominant and recessive.

**Figure S2**. Powers and type I errors for Scenarios 1 to 3, windows defined by BP. Shown are bar plots of the calculated powers (for *P*-values 10^−8^, 10^−6^ and 10^−4^) and type I errors (for *p*-values 10^−4^,  and 10^−3^) for Scenarios 1 to 3 for imputation (Imp), haplotype analysis (Hap), single-SNP logistic regression (LR) and the AI test with different SNP window sizes defined by base pair position on the genome. The standard multiplicative model and correlation metrics were used in the AItest.

**Figure S3**. Powers for dominant and recessive models. Shown are bar plots of the calculated powers for *p*-values 10^−8^, 10^−6^ and 10^−4^. Plots on the left show data simulated under a dominant model and plots on the right show data simulated under a recessive model. Rows 1-3 show Scenarios 1-3 respectively.

**Figure S4**. Manhattan plots of results from genome-wide imputation and AI in the Gambian case/control and trio (case/pseudocontrol) datasets. Pink and green dots denote imputation results (Imp) for odd and even chromosomes respectively. Black dots denote AIresults.

**Figure S5**. Manhattan plots of results from haplotype analysis of the Gambian case/control and trio (case/pseudocontrol) datasets. SNP index refers to the middle SNP of a 5-SNP haplotype. Pink and green dots denote results for odd and even chromosomes respectively.
