# Supplementary material for: Imputation Without Doing Imputation: A New Method for the Detection of Non-Genotyped Causal Variants
Source: Genet Epidemiol. 2014 Feb 17;38(3):173–90. doi: 10.1002/gepi.21792 (PMC4150535; doi:10.1002/gepi.21792)
Supplement: Supplementary file 1 — Figure S1. Plots of the correlation metrics used in the AItest to choose a partner SNP. The solid black line shows the fitted curve which maps the anchor-partner SNP correlation to a score in the interval [0, 100]. The black dots show the evaluated correlation values from simulations. Both the dots and fitted curve have been rescaled so the peak of the curve is at value 100. From left to right the correlation metrics are defined for causal variants that are: multiplicative, dominant and recessive. [file gepi0038-0173-sd1.pdf]

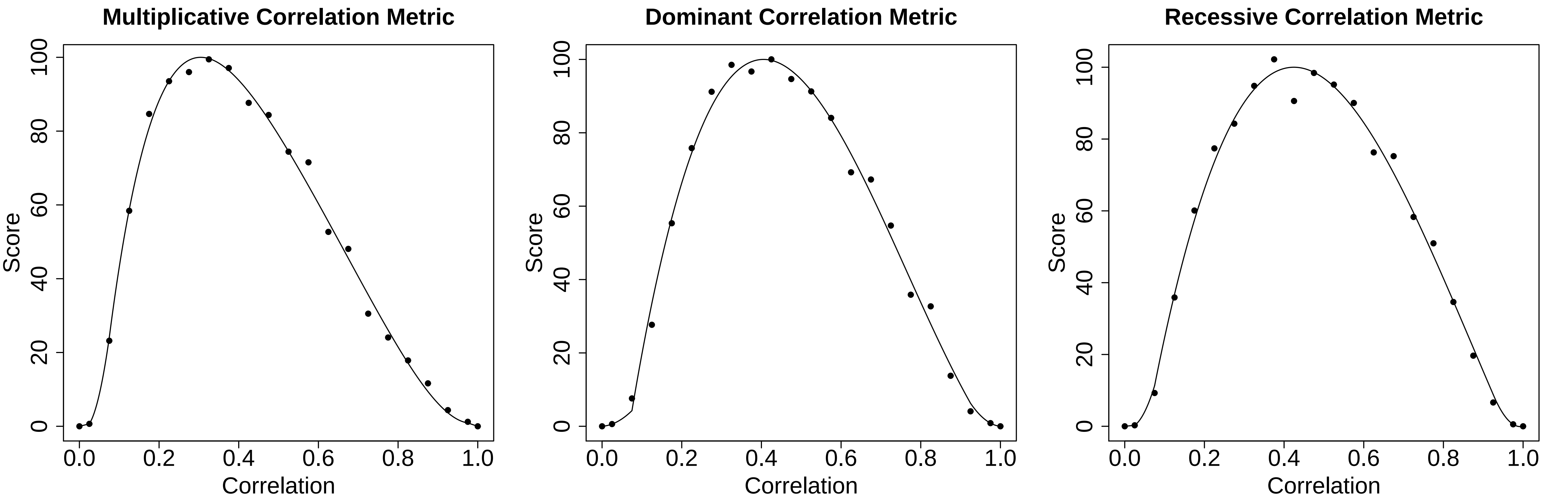

Multiplicative Correlation Metric
Dominant Correlation Metric
Recessive Correlation Metric
Score
Correlation
0
20
40
60
80
100
0.0
0.2
0.4
0.6
0.8
1.0
