# Supplementary figures and images for: Imputation Without Doing Imputation: A New Method for the Detection of Non-Genotyped Causal Variants
Source: Genet Epidemiol. 2014 Feb 17;38(3):173–90. doi: 10.1002/gepi.21792 (PMC4150535; doi:10.1002/gepi.21792)

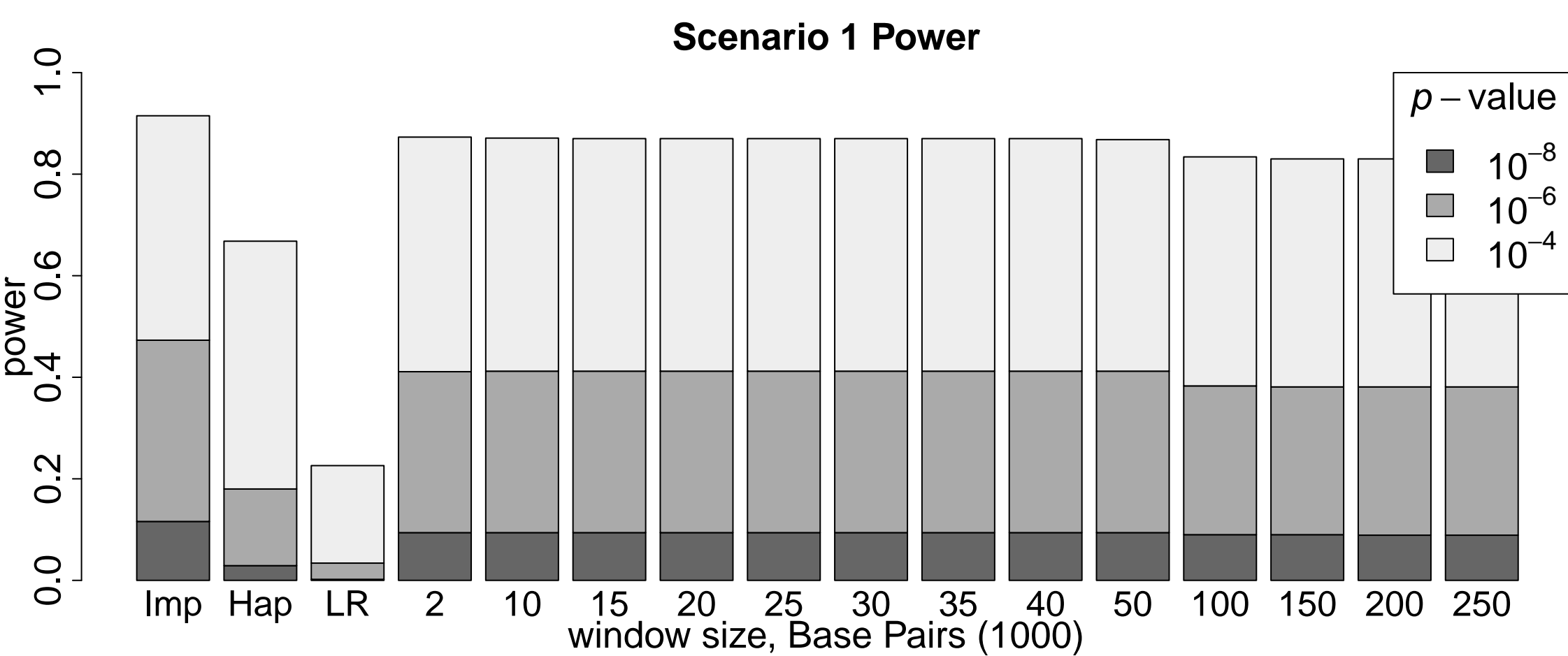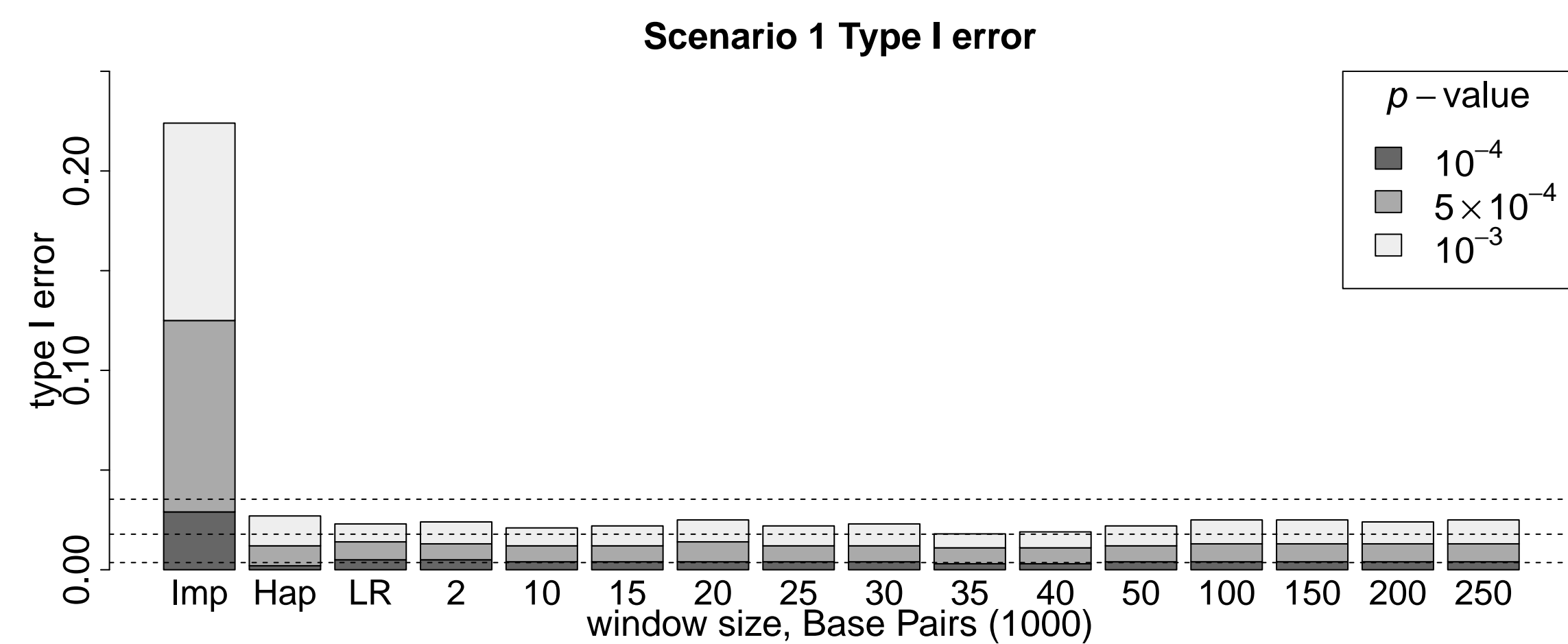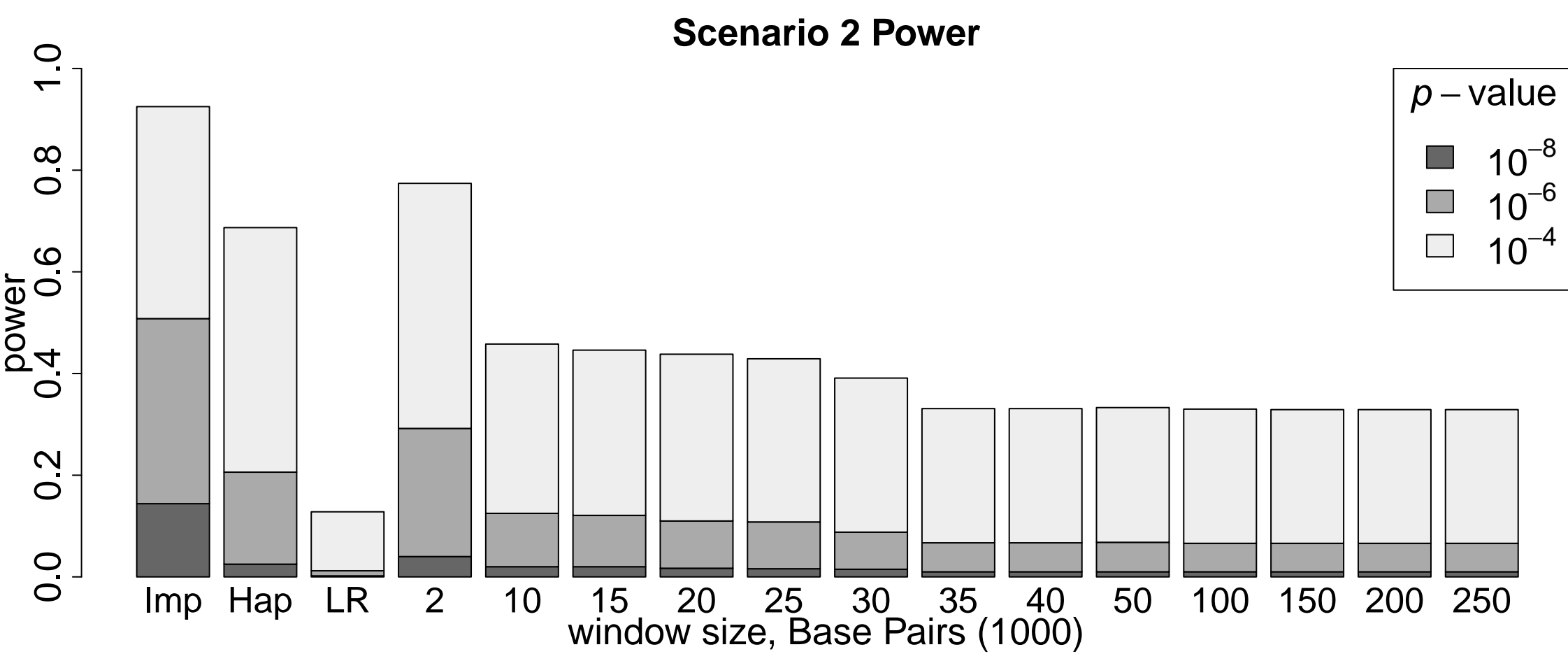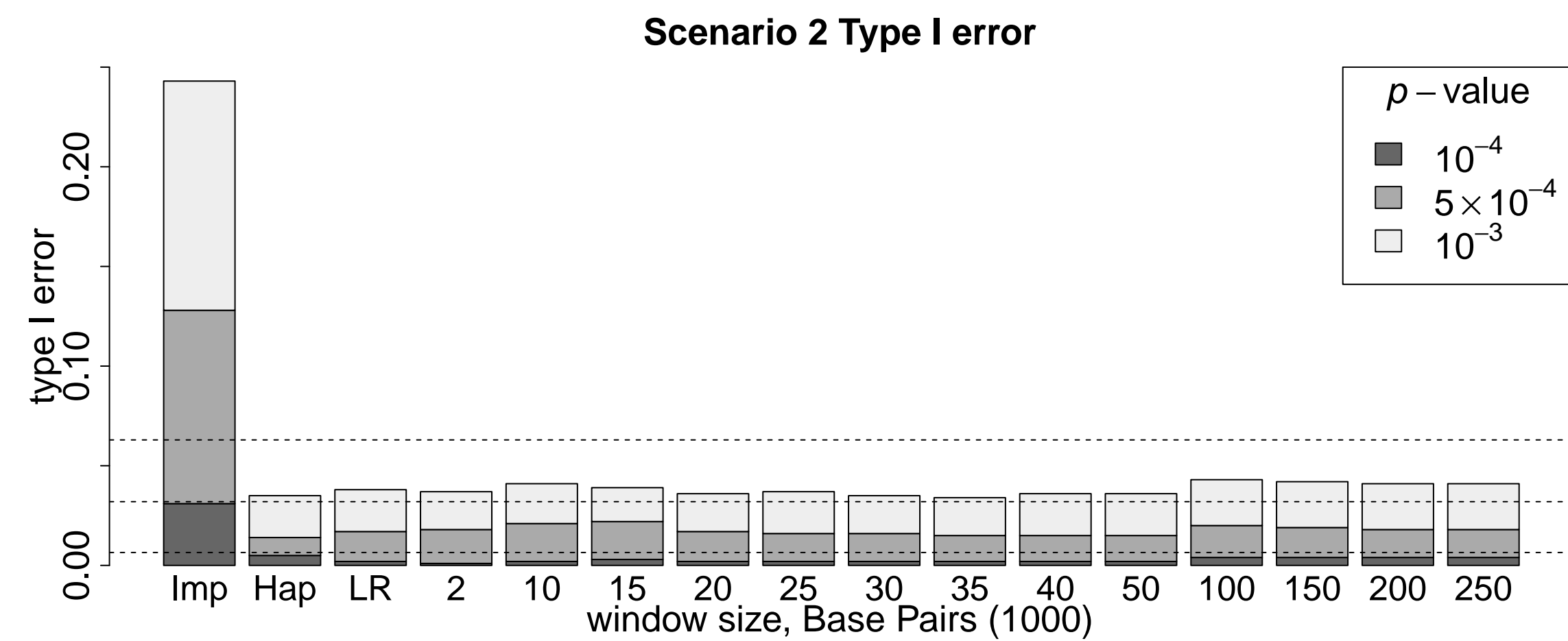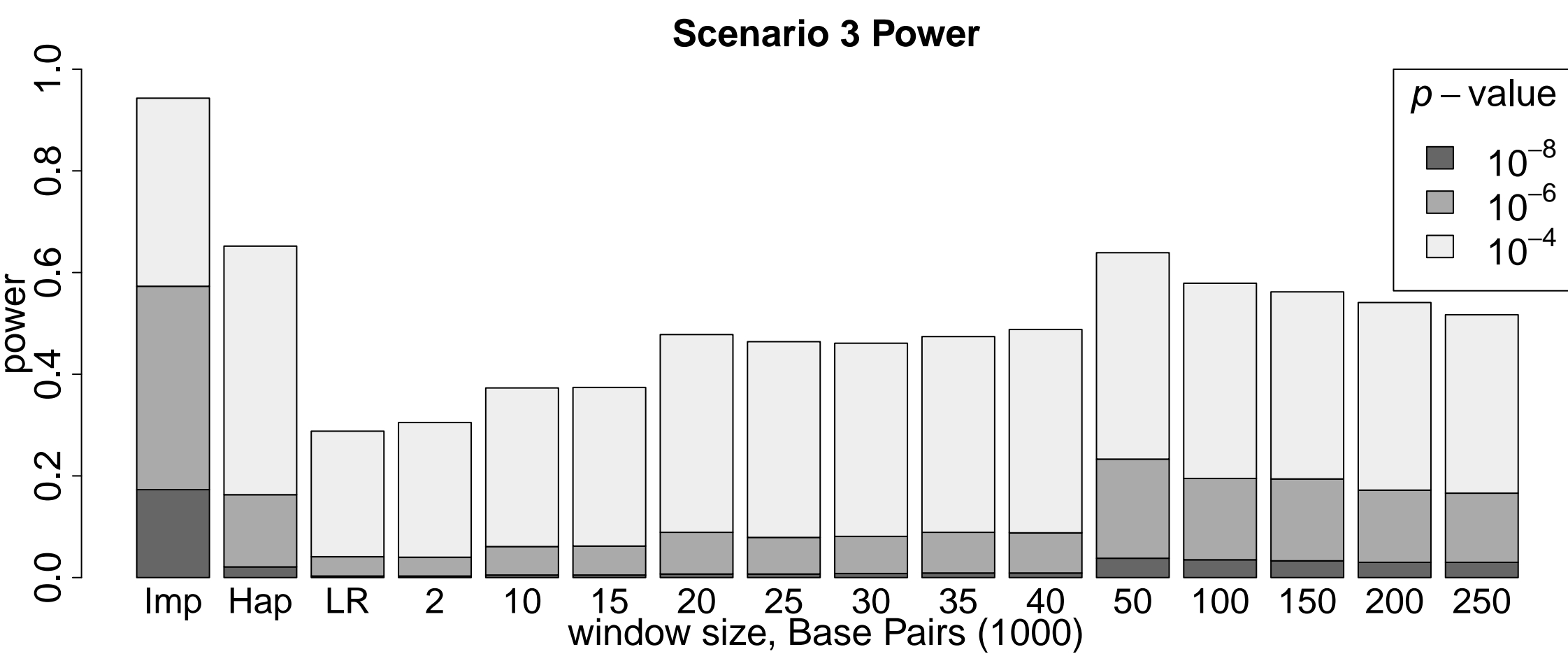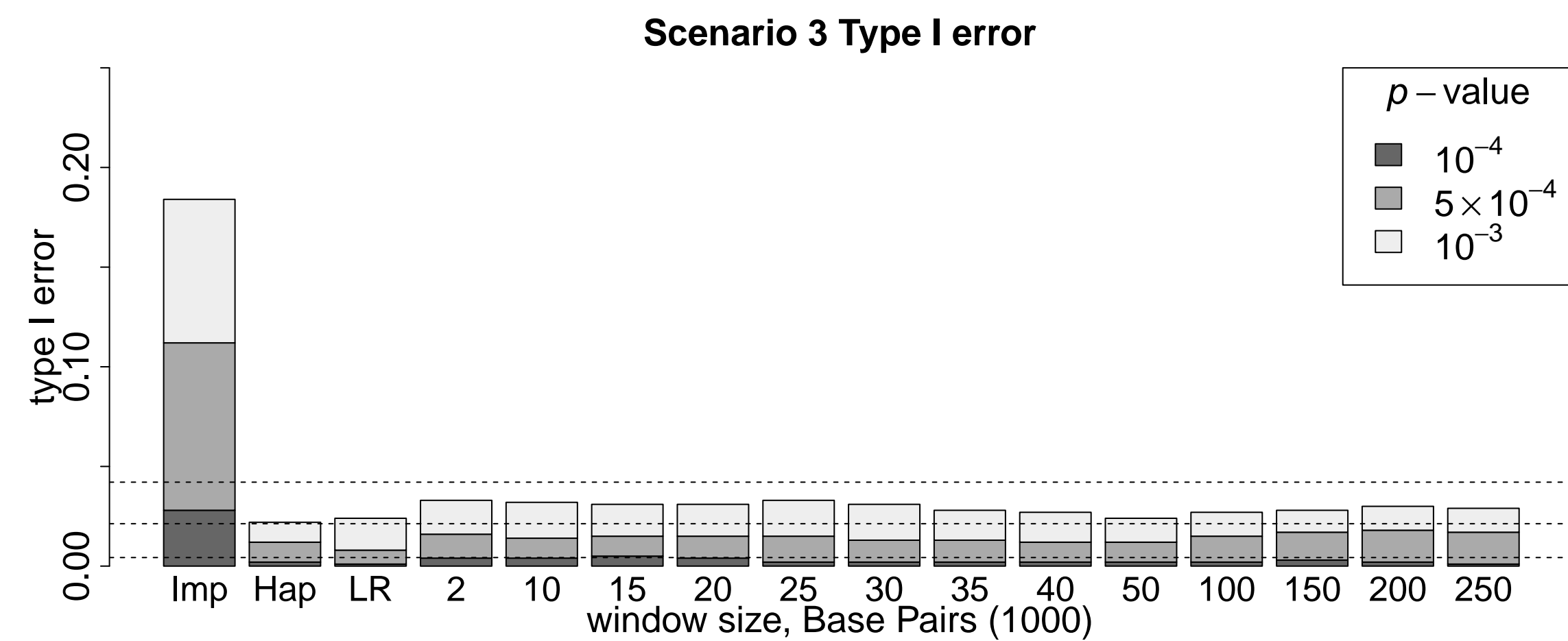

Supplement: Supplementary file 2 — Figure S2. Powers and type I errors for Scenarios 1 to 3, windows defined by BP. Shown are bar plots of the calculated powers (for P-values 10−8, 10−6 and 10−4) and type I errors (for p-values 10−4, and 10−3) for Scenarios 1 to 3 for imputation (Imp), haplotype analysis (Hap), single-SNP logistic regression (LR) and the AI test with different SNP window sizes defined by base pair position on the genome. The standard multiplicative model and correlation metrics were used in the AItest. [file gepi0038-0173-sd2.pdf]

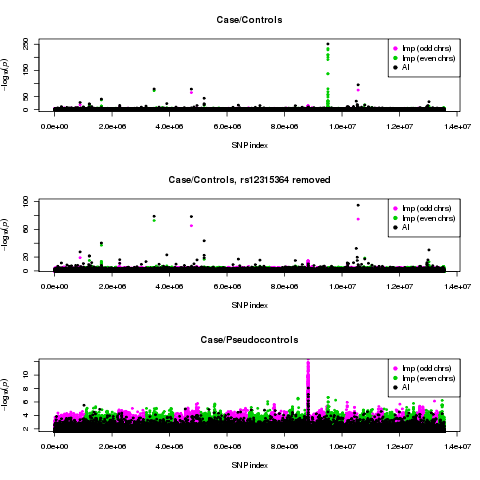

Supplement: Supplementary file 4 — Figure S4. Manhattan plots of results from genome-wide imputation and AI in the Gambian case/control and trio (case/pseudocontrol) datasets. Pink and green dots denote imputation results (Imp) for odd and even chromosomes respectively. Black dots denote AIresults. [file gepi0038-0173-sd4.png]

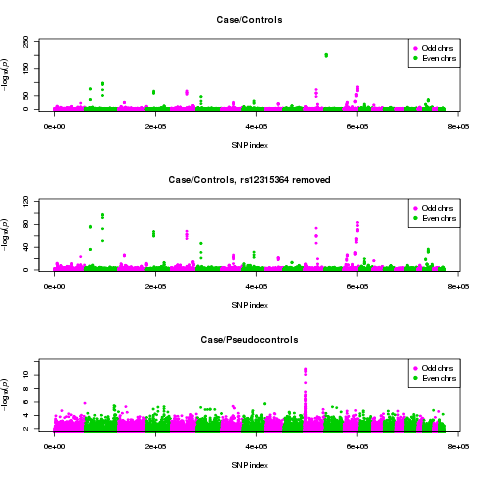

Supplement: Supplementary file 5 — Figure S5. Manhattan plots of results from haplotype analysis of the Gambian case/control and trio (case/pseudocontrol) datasets. SNP index refers to the middle SNP of a 5-SNP haplotype. Pink and green dots denote results for odd and even chromosomes respectively. [file gepi0038-0173-sd5.png]
