# Supplementary material for: Imputation Without Doing Imputation: A New Method for the Detection of Non-Genotyped Causal Variants
Source: Genet Epidemiol. 2014 Feb 17;38(3):173–90. doi: 10.1002/gepi.21792 (PMC4150535; doi:10.1002/gepi.21792)
Supplement: Supplementary file 3 — Figure S3. Powers for dominant and recessive models. Shown are bar plots of the calculated powers for p-values 10−8, 10−6 and 10−4. Plots on the left show data simulated under a dominant model and plots on the right show data simulated under a recessive model. Rows 1-3 show Scenarios 1-3 respectively. [file gepi0038-0173-sd3.pdf]

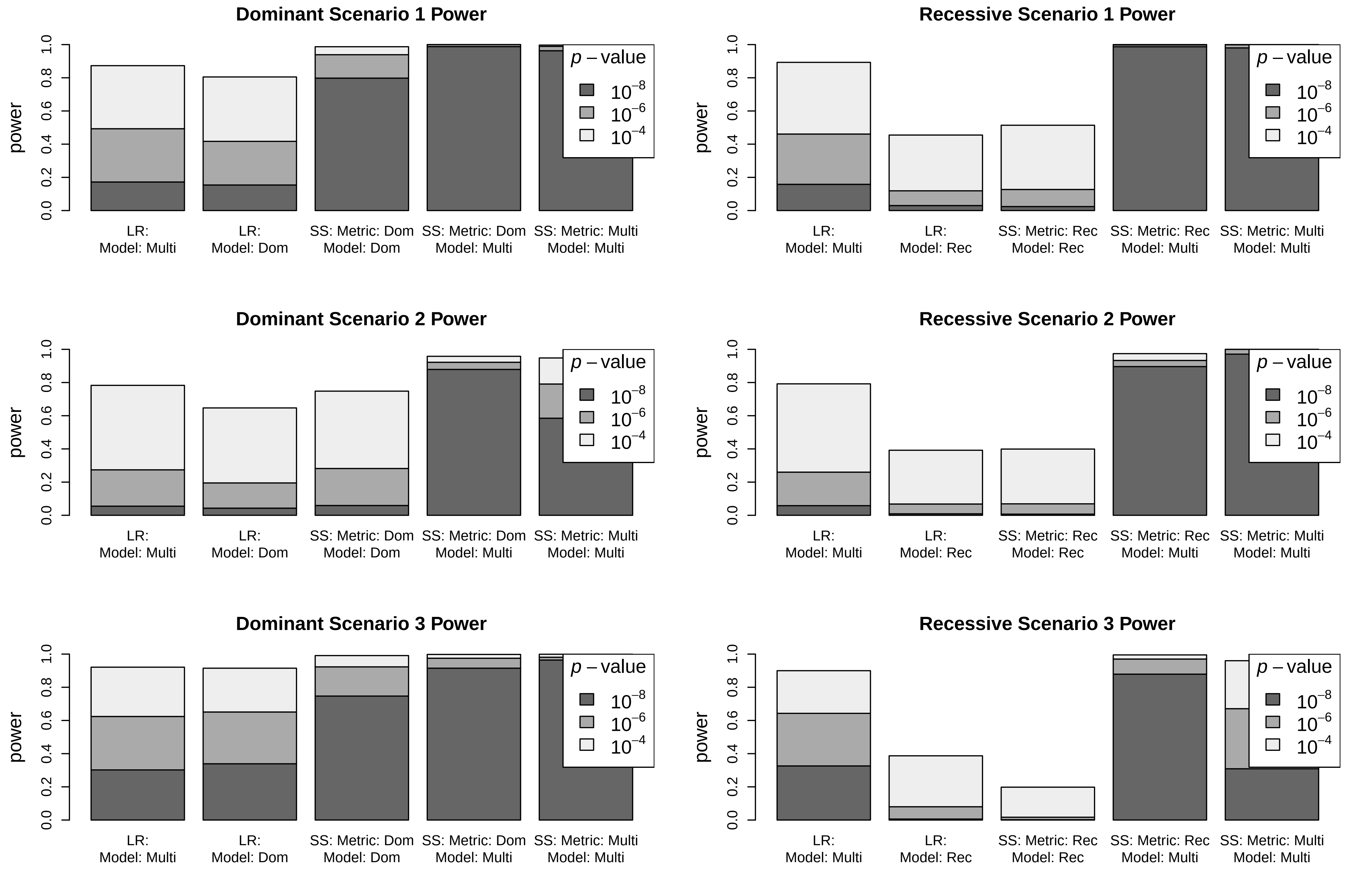
Dominant Scenario 1 Power
Recessive Scenario 1 Power
Dominant Scenario 2 Power
Recessive Scenario 2 Power
Dominant Scenario 3 Power
Recessive Scenario 3 Power
power
0.0
0.2
0.4
0.6
0.8
1.0
p – value
10^{-8}
10^{-6}
10^{-4}
LR: Model: Multi
LR: Model: Dom
SS: Metric: Dom Model: Dom
SS: Metric: Dom Model: Multi
SS: Metric: Multi Model: Multi
LR: Model: Rec
SS: Metric: Rec Model: Rec
SS: Metric: Rec Model: Multi
